# Infection with flaviviruses requires BCLX_L_ for cell survival

**DOI:** 10.1371/journal.ppat.1007299

**Published:** 2018-09-27

**Authors:** Tatsuya Suzuki, Toru Okamoto, Hiroshi Katoh, Yukari Sugiyama, Shinji Kusakabe, Makoto Tokunaga, Junki Hirano, Yuka Miyata, Takasuke Fukuhara, Masahito Ikawa, Takashi Satoh, Sachiyo Yoshio, Ryosuke Suzuki, Masayuki Saijo, David C. S. Huang, Tatsuya Kanto, Shizuo Akira, Yoshiharu Matsuura

**Affiliations:** 1 Department of Molecular Virology, Research Institute for Microbial Diseases, Osaka University, Osaka, Japan; 2 Department of Virology III, National Institute of Infectious Diseases, Tokyo, Japan; 3 Department of Experimental Genome Research, Research Institute for Microbial Diseases, Osaka University, Osaka, Japan; 4 Department of Host Defense, Research Institute for Microbial Diseases, Osaka University, Osaka, Japan; 5 The Research Center for Hepatitis and Immunology, National Center for Global Health and Medicine, Chiba, Japan; 6 Department of Virology II, National Institute of Infectious Diseases, Tokyo, Japan; 7 Department of Virology I, National Institute of Infectious Diseases, Tokyo, Japan; 8 Walter and Eliza Hall Institute of Medical Research, Parkville, Victoria, Australia; 9 Department of Medical Biology, University of Melbourne, Parkville, Victoria, Australia; The University of Chicago, UNITED STATES

## Abstract

BCL2 family proteins including pro-survival proteins, BH3-only proteins and BAX/BAK proteins control mitochondria-mediated apoptosis to maintain cell homeostasis via the removal of damaged cells and pathogen-infected cells. In this study, we examined the roles of BCL2 proteins in the induction of apoptosis in cells upon infection with flaviviruses, such as Japanese encephalitis virus, Dengue virus and Zika virus. We showed that survival of the infected cells depends on BCLX_L_, a pro-survival BCL2 protein due to suppression of the expression of another pro-survival protein, MCL1. Treatment with BCLX_L_ inhibitors, as well as deficient BCLX_L_ gene expression, induced BAX/BAK-dependent apoptosis upon infection with flaviviruses. Flavivirus infection attenuates cellular protein synthesis, which confers reduction of short-half-life proteins like MCL1. Inhibition of BCLX_L_ increased phagocytosis of virus-infected cells by macrophages, thereby suppressing viral dissemination and chemokine production. Furthermore, we examined the roles of BCLX_L_ in the death of JEV-infected cells during *in vivo* infection. Haploinsufficiency of the BCLX_L_ gene, as well as administration of BH3 mimetic compounds, increased survival rate after challenge of JEV infection and suppressed inflammation. These results suggest that BCLX_L_ plays a crucial role in the survival of cells infected with flaviviruses, and that BCLX_L_ may provide a novel antiviral target to suppress propagation of the family of *Flaviviridae* viruses.

## Author summary

The genus *Flavivirus* including Japanese encephalitis virus, Dengue virus, and Zika virus all of which are mosquito-borne human pathogen and cause serious diseases in humans. Therefore, the development of effective vaccines and antivirals against several flaviviruses is still needed. BCL2 family proteins control mitochondria-mediated apoptosis to maintain cell homeostasis via the removal of damaged cells and pathogen-infected cells, deregulation of which leads to severe diseases including cancer and autoimmune diseases. Here, we showed that BCLX_L_ is a critical cell survival factor during infection with flaviviruses, and that inhibition of BCLX_L_ by treatment with BH3 mimetics restricts the production of infectious particles and the expression of chemokines *in vitro* and *in vivo*. Inhibition of BCLX_L_ induces apoptosis in cells infected with flaviviruses and these cells are quickly removed by engulfment of phagocytes, which leads to inhibition of virus dissemination without any inflammatory reaction. Based on these data, BCLX_L_ would appear to be a suitable target for the development of novel antivirals against a broad range of flavivirus infections.

## Introduction

The genus *Flavivirus* belongs to the family *Flaviviridae* and includes the Japanese encephalitis virus (JEV), Dengue virus (DENV), West Nile virus (WNV) and Zika virus (ZIKV), all of which are mosquito-borne human pathogens [1,2]. The flaviviruses are internalized into dendritic cells, such as Langerhans cells [3], and keratinocytes in skin [4] through mosquito bites. Dendritic cells that are thus infected with flaviviruses are activated and migrate into the lymph nodes, facilitating viral spread into peripheral tissues [5] and the induction of visceral and/or central nervous system diseases. DENV infection may generate dengue haemorrhagic fever and dengue shock syndrome [6], while infection with either WNV or JEV can cause severe encephalitis [7,8]. Recently, an outbreak of ZIKV infection in Brazil was linked to birth defects, including microcephaly, and to Guillain-Barre syndrome in adults [9,10]. Therefore, the development of effective vaccines and antiviral drugs against the family of flaviviruses is an urgent need.

Infection with flaviviruses induces apoptosis in cells by the production of reactive oxidative species, unfolded protein responses, and viral proteins [11], in addition to other types of cell death, such as necrosis and pyroptosis. Receptor-interacting serine/threonine-protein kinase-3 (RIPK3), a central player of necroptosis, has been shown to play an additional role during virus infection [12]. Infection with either DENV or hepatitis C virus (HCV) induces interleukin-1β production and pyroptosis [13]. The biological significance of pyroptosis in the pathogenicity of flavivirus infection remains largely unknown [14].

Apoptosis is a type of programmed cell death that removes excess cells during development, as well as during routine homeostasis [15–17]. B-cell lymphoma-2 (BCL2) family proteins play central roles in mitochondria-mediated apoptosis [18]. BCL2 family proteins have been divided into three groups: BH3-only proteins, BAX/BAK proteins and pro-survival proteins. Once an apoptotic stimulus such as DNA damage occurs, BH3-only proteins, such as BIM, BID, PUMA, BAD and NOXA, are activated and directly or indirectly activate BAX and BAK. Activated BAX/BAK forms homo-oligomers that permeabilize mitochondria and allow the release of pro-apoptotic factors such as cytochrome c, which promotes activation of caspases [19]. Pro-survival proteins, such as BCL2, BCLW, BCLX_L_, MCL1 and A1, sequester BH3-only proteins to prevent self-activation of BAX/BAK by interaction with the BH3 domains of BAX/BAK [20]. In a notable extension to cancer therapy, small molecule inhibitors have been developed, including ABT-737, ABT-263 (navitoclax, an orally available clinical derivative of ABT-737), ABT-199 (venetoclax) and A-1331852. ABT-199 and A-1331852 target BCL2 and BCLX_L_, respectively, while ABT-737 and ABT-263 target BCL2, BCLX_L_ and BCLW. ABT-199 was approved by the United States Food and Drug Administration for the treatment of 17-p deleted chronic lymphocytic leukaemia (CLL) [21], supporting the therapeutic use of induction of cell death by mitochondria-mediated apoptosis to eliminate unnecessary cells.

In this study, we showed that cells infected with flaviviruses including JEV, DENV and ZIKV specifically induced apoptosis by inhibition of BCLX_L_ through the suppression of MCL1 expression. Furthermore, we showed that host protein synthesis is impaired in cells upon infection with flaviviruses, leading to the decay of labile proteins such as MCL1. Treatment with inhibitors for BCLX_L_, as well as deficiency of BCLX_L_ gene, induced BAX/BAK-dependent apoptosis in the infected cells. Finally, engulfment of virus-infected cells by macrophages was significantly enhanced by the inhibition of BCLX_L_ without an increase of the production of pro-inflammatory cytokines, which led to the suppression of viral dissemination.

## Results

### Inhibition of BCLX_L_ accelerates cell death upon infection with flaviviruses

**Fig 1:**
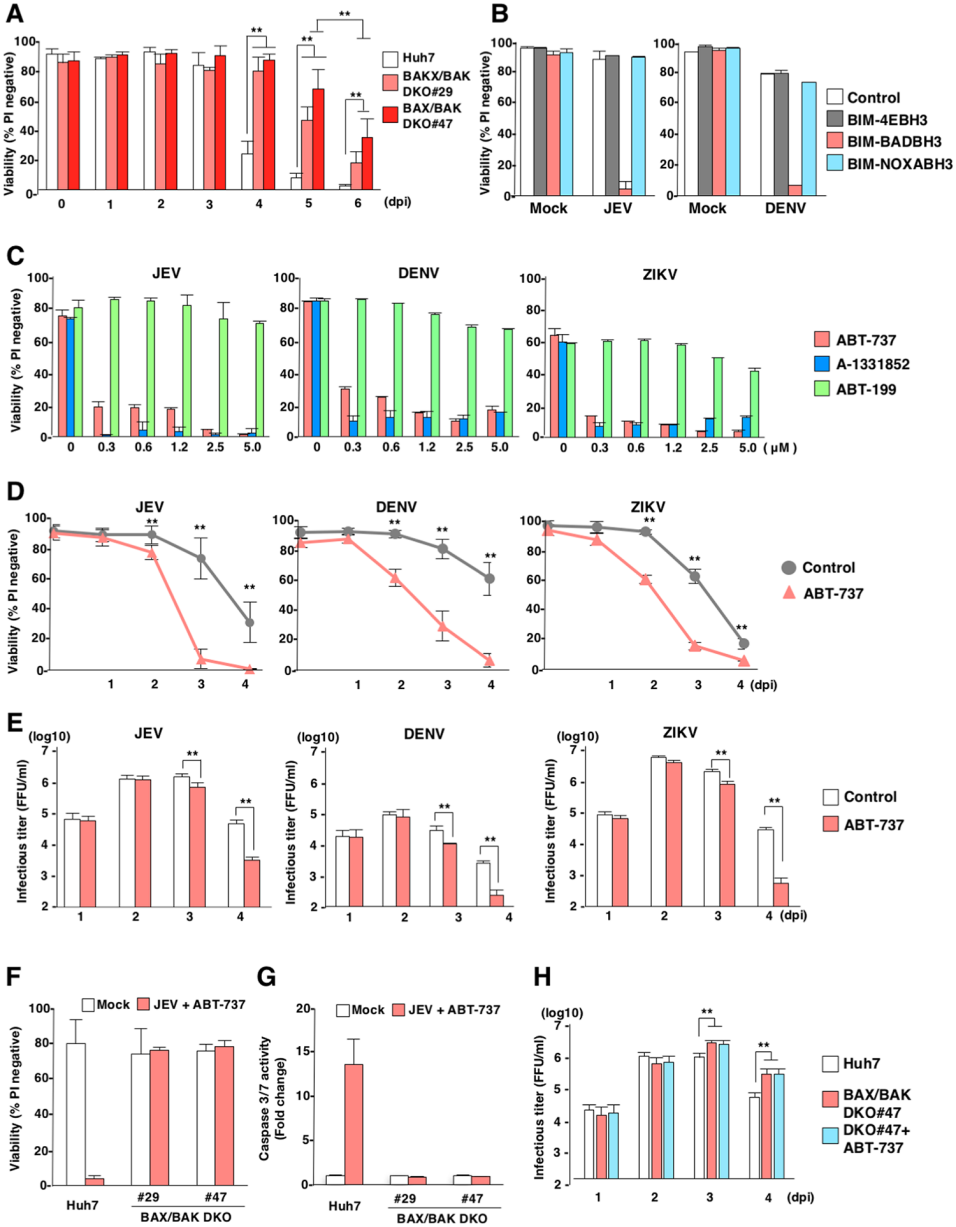
BCLX_L_ participates in the survival of cells infected with flaviviruses. (A) Parental, BAX/BAKDKO (two clones, #29 and #47) Huh7 cell lines were infected with JEV (MOI = 5). Cell viability was assessed at the indicated time points. (B) Ectopic expression of BIM-BADBH3 accelerates flavivirus-induced cell death. Parental, BIM-4E, BIM-BADBH3, and BIM-NOXABH3 Huh7 cells were infected with JEV (MOI = 5; left panel) or DENV (MOI = 3; right panel). Cell viability was assessed at 3 days post-infection. (C) Huh7 cells infected with either JEV (MOI = 5; left panel), DENV (MOI = 3; middle panel) or ZIKV (MOI = 1; right panel) were incubated at 37°C for 2 h; then, culture medium was replaced with medium containing either of the following: ABT-737, A-1331852, or ABT-199, at the indicated concentration. Cell viability was assessed at 3 days post-infection. (D) Huh7 cells infected with JEV (MOI = 5), DENV (MOI = 3) or ZIKV (MOI = 1), and treated with ABT-737 (1 μM). Cell viability was assessed at the indicated time points. (E) Huh7 cells infected with either JEV (MOI = 5; left panel), DENV (MOI = 3; middle panel) or ZIKV (MOI = 1; right panel) were treated with ABT-737 (1 μM). Infectious titers in the culture supernatants were determined by focus-forming assay at the indicated time points. (F,G) Parental and BAX/BAKDKO (two clones, #29 and #47) Huh7 cells were infected with JEV (MOI = 5) and treated with ABT-737 (1 μM). Cell viability was assessed at 3 days post-infection (F). Caspase 3/7 activity was measured at 2 days post-infection (G). (H) Parental and BAX/BAKDKO (one clone, #47) Huh7 cells were infected with JEV (MOI = 5) or infected with JEV and treated with ABT-737 (1 μM). Infectious titers in the culture supernatants were determined at the indicated time points. The data in Figure 1 represent the mean ± SD of two (B,C,F,G) or three (A,D,E,H) independent experiments. Significant differences were determined using Student’s *t*-test and are indicated by asterisks (*P<0.05) and double asterisks (**P<0.01).

Firstly, to examine the roles of BAX/BAK-dependent apoptosis during flavivirus-infected cells, we generated BAX and BAK double-knockout (BAX/BAKDKO) Huh7 cell lines (clones #29 and #47) and infected with Japanese encephalitis virus (JEV) (S1A Fig). Although Huh7 cells induced cell death at 4 days post-infection with JEV, BAX/BAKDKO cells exhibited a complete resistant to mitochondria mediated apoptosis [22], suggesting that cells infected with JEV induce mitochondria-mediated apoptosis at 4 days post-infection. However, BAX/BAKDKO cells also exhibited cell death at 5 and 6 days post-infection. The BAX/BAK-independent cell death might be induced by necrosis or pyroptosis rather than apoptosis as previously reported (Fig 1A) [12].

Recent clinical studies showed that survival of chronic lymphocytic leukaemia (CLL) is largely dependent on BCL2 [21], and that loss of a single *Bclx* allele attenuates MYC-induced lymphoma *in vivo* [23]. In addition, selective inhibition of MCL1 has been shown to induce apoptosis in acute myelogenous leukaemia cell lines without signs of toxicity to normal human haematopoietic progenitor cells [24]. It had been reported that cellular stress such as ER stress, DNA damage and innate immune response affects on the expression of BCL2 protein family [25–27]. Therefore, we speculated that virus infection alters the expression of BCL2 proteins through the induction of cellular stress. BH3-only proteins are known to exhibit specific interactions with pro-survival BCL2 proteins, including BCL2, BCLX_L_, BCLW and MCL1 [28]. BIM and PUMA bind to all pro-survival BCL2 proteins; BAD binds selectively to BCL2, BCLX_L_ and BCLW; and NOXA binds specifically to MCL1 (S1B Fig). To investigate the roles of pro-survival BCL2 proteins in cells infected with flaviviruses, we generated Huh7 cell lines stably expressing a BIM-mutant possessing either a binding-defective BH3 domain (BIM-4EBH3), the BH3 domain from BAD (BIM-BADBH3), or the BH3 domain from NOXA (BIM-NOXABH3) (S1C and S1D Fig). Although lentivirus-induced expression of BIM-NOXABH3 or BIM-BADBH3 specifically killed Huh7 cells expressing BIM-BADBH3 or BIM-NOXABH3, respectively, parental and Huh7 cells expressing BIM-4EBH3 were resistant to killing through the expression of BIM-mutants (S1E Fig). Although no cell death was observed in parental Huh7 cells, or in cells expressing either BIM-4EBH3 or BIM-NOXABH3, upon infection with JEV and DENV, cells expressing BIM-BADBH3 showed a significant reduction in viability at 3 days-post infection (Fig 1B).

To assess which pro-survival BCL2 proteins were responsible for the survival of cells infected with flaviviruses, the effects of BH3 mimetics on flavivirus-infected cells were determined. No cytotoxicity was observed in Huh7 cells solely treated with BH3 mimetics at 3days post treatment (S1F Fig). Cells infected with JEV, DENV and ZIKV, and treated with either ABT-737 [29] (targeting BCL2, BCLW and BCLX_L_) or A-1331852 [30] targeting BCLX_L_ exhibited cell death at 3 days-post infection. In contrast, infected cells treated with ABT-199 [31] (targeting BCL2) showed no cell death (Fig 1C and S1B Fig). Treatment of ABT-737 (1 μM) significantly accelerated JEV, DENV and ZIKV induced cell death (Fig 1D). Next, to examine the roles of BCLX_L_ inhibition on infectious virus production, we quantified viral titres by using a focus-forming assay. Treatment with ABT-737 (1 μM) exhibited no effect on the infectious particle production of JEV, DENV and ZIKV until 2-days post-infection, but significantly reduced the infectious titers at 3 and 4 days post-infection (Fig 1E), suggesting that induction of cell death by the treatment with ABT-737 in viral infected cells reduces infectious virus production. We next determined whether ABT-737 treatment induces BAX/BAK-dependent cell death upon infection with flaviviruses. Although parental Huh7 cells exhibited cell death upon JEV infection and treatment with ABT-737, which activated caspase 3/7, BAX/BAKDKO cells were resistant to cell death under the same conditions, and no activation of caspase 3/7 was detected (Fig 1F and 1G). In addition, release of cytochrome c was observed in parental Huh7 cells infected with JEV and treated with ABT-737, but not in BAX/BAKDKO cells (S1G Fig). Although no increase of infectious particle production was observed in BAX/BAKDKO cells until 2-days post-infection, three to ten times of increase of infectious titer compared to parental Huh7 cells was observed in BAX/BAKDKO cells at 3 and 4 days post-infection (Fig 1H). Collectively, these results suggest that cell death is induced upon infection with JEV and treatment with ABT-737 through a BAX/BAK-dependent pathway.

### BCLX_L_ plays crucial roles in cell survival upon infection with flaviviruses

**Fig 2:**
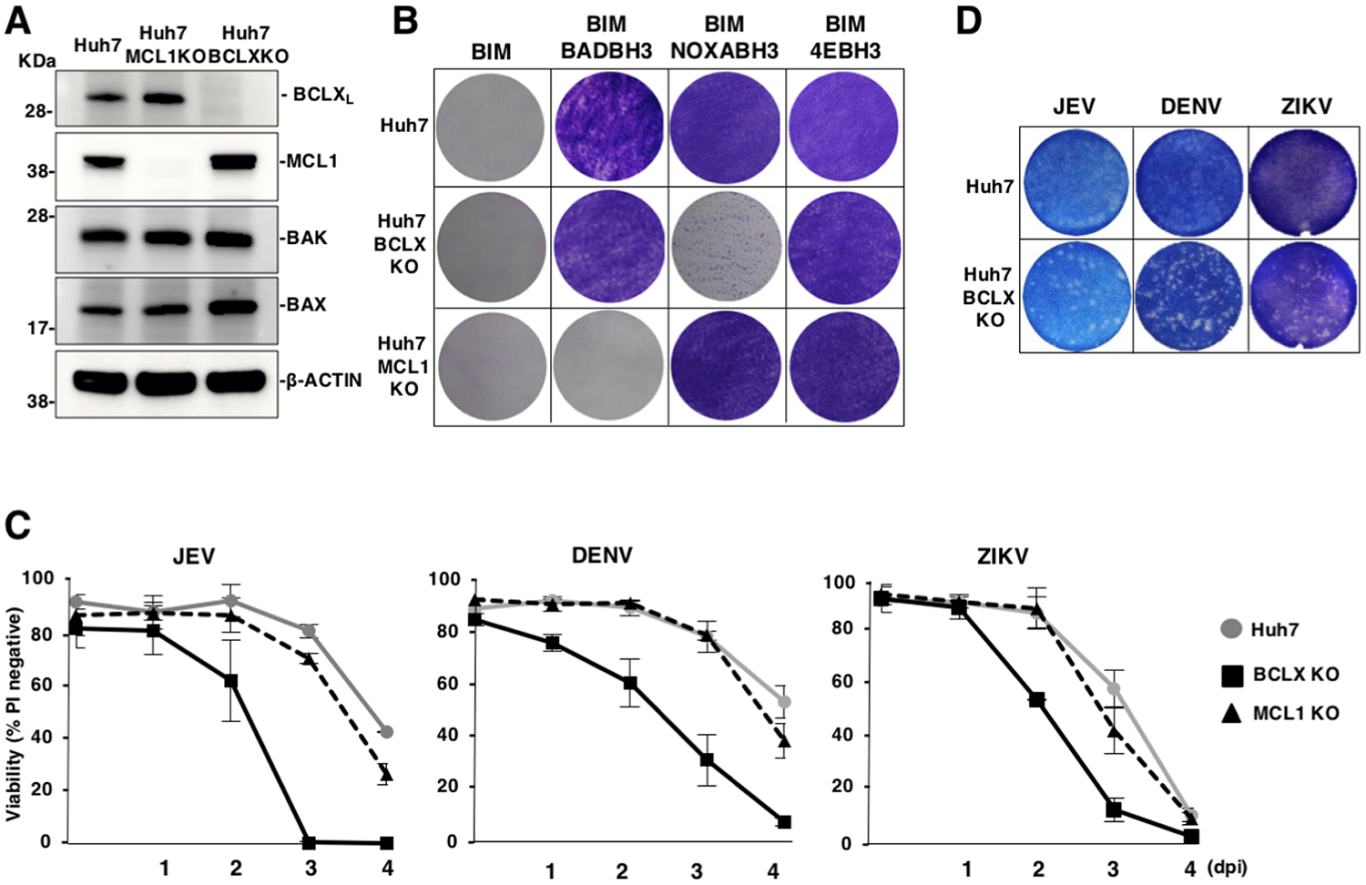
BCLX_L_ is a critical survival factor against flavivirus infection. (A) Expression of BCLX_L_, MCL1, BAK, and BAX in parental, BCLXKO and MCL1KO Huh7 cells was determined by immunoblotting. (B) Parental, BCLXKO and MCL1KO Huh7 cell lines were infected with lentiviruses expressing the indicated BIM-mutants, then cultured in the presence of puromycin. Remaining adherent cells were visualized by Giemsa staining. (C) Deficiency of BCLX_L_ accelerates cell death upon infection with flaviviruses. Parental, BCLXKO and MCL1KO Huh7 cell lines were infected with JEV (MOI = 5), DENV (MOI = 3) or ZIKV (MOI = 1). Cell viability was assessed at the indicated time points. (D) Parental and BCLXKO Huh7 cell lines were infected with JEV (100 FFU), DENV (100 FFU) or ZIKV (500 FFU). At 2 days post-infection, the cells were stained with methylene blue or Giemsa staining. Images are representative of two independent experiments performed with a culture representative of each cell line (A,B,D). The data represent the mean ± SD of three independent experiments performed with a culture representative of each cell line (C).

To confirm the BCLX_L_-dependent survival of cells upon infection with flaviviruses, we generated Huh7 cell lines that were deficient in either BCLX_L_ or MCL1 (BCLXKO or MCL1KO) (Fig 2A). Expression of BIM-BADBH3 and BIM-NOXABH3 killed MCL1KO and BCLXKO cells, respectively, while expression of BIM-4EBH3 conferred resistance to both parental and KO cells (Fig 2B). Among the pro-survival proteins of BCL2 protein family, BCLX and MCL1 redundantly suppress activation of both of BAX and BAK. On the other hands, BCL2 and BCLW had been reported to suppress BAX but not BAK [32–36]. In addition, deficiency of BCLW in mice developed normally except spermatogenesis in male mice, suggesting that BCLW is important for apoptosis in testis [37]. To examine the redundancy of BCL2 proteins, we treated parental, MCL1KO and BCLXKO Huh7 cells with either A-1331852, BCLX-specific inhibitor or S63845, MCL1 selective inhibitor [24]. MCL1KO Huh7 cells treated with A-1331852 (1 μM) but not with S63845 (1 μM) and BCLXKO Huh7 cells treated with S63845 (1 μM) but not with A-1331852 (1 μM) induced cell death, while parental Huh7 cells are resistant to the treatment with either compounds but induced cell death by the treatment with both compounds (S2A Fig). These data suggest that BCLX and MCL1 redundantly suppress activation of BAX and BAK. The viabilities of BCLXKO cells upon infection with JEV, DENV or ZIKV were consistently lower than those of parental and MCL1KO Huh7 cells (Fig 2C), and we confirmed the induction of cell death upon infection with JEV in other independently established BCLXKO Huh7 cell lines (S2B and S2C Fig). Next, we determined the effect of BCLX inhibition on plaque formation. Furthermore, Huh7 cells did not form any plaque upon infection with JEV, DENV and ZIKV, however, BCLXKO Huh7 cells exhibited formation of clear plaques upon infection with these viruses (Fig 2D). Taken together, these results indicate that apoptosis is induced in BCLX-deficient cells upon infection with flaviviruses.

### Expression of MCL1 is impaired in cells infected with flaviviruses

**Fig 3:**
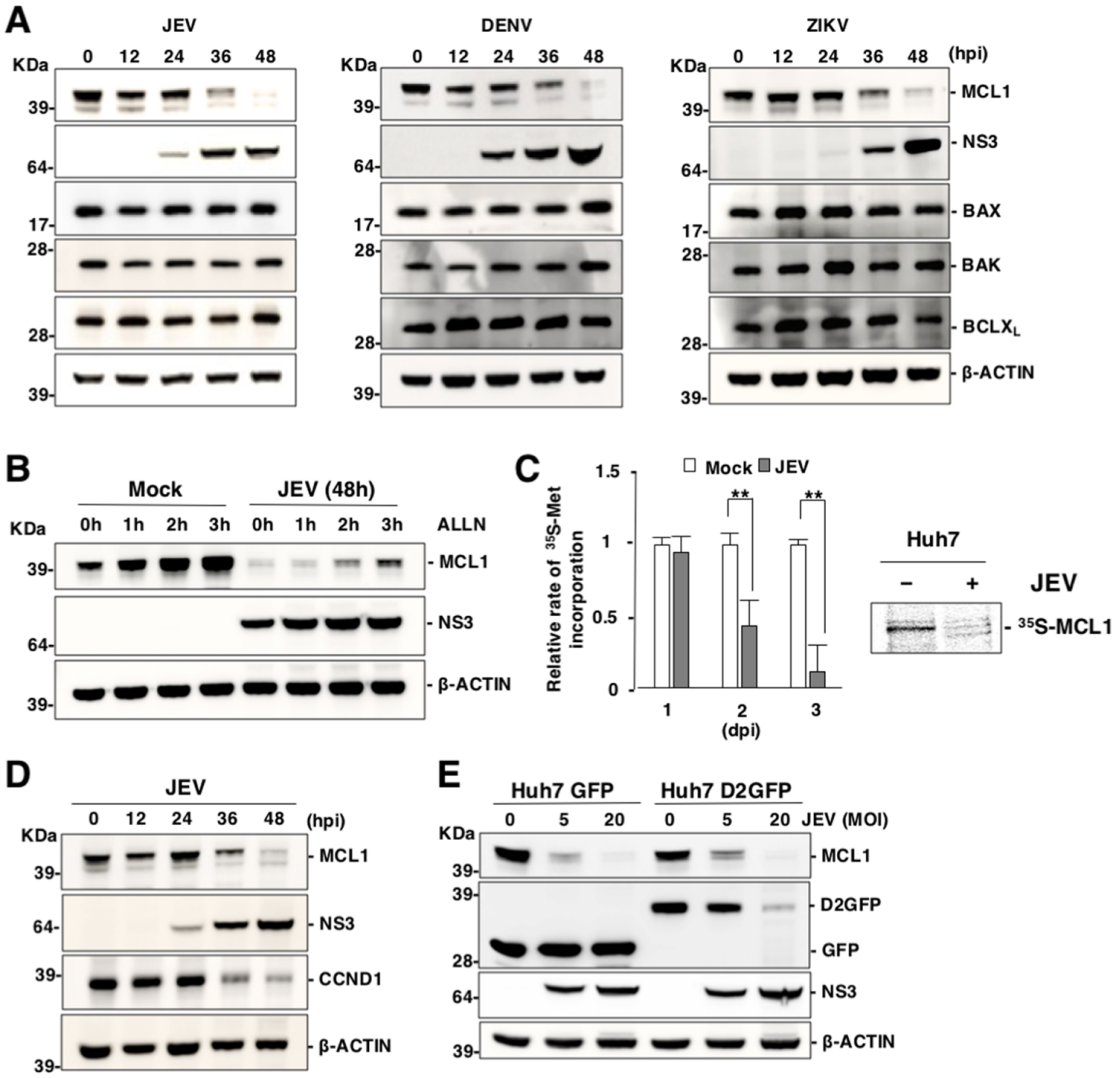
Expression of MCL1 is impaired in cells infected with flaviviruses. (A) Expression of MCL1 is decreased in cells infected with flaviviruses. Huh7 cells were infected with either JEV (MOI = 5), DENV (MOI = 3) or ZIKV (MOI = 1), then incubated with the caspase inhibitor, QVD-OPH (20 μM). Expression of the indicated proteins was assessed by immunoblotting. (B) Huh7 cells were infected as described above were incubated with QVD-OPH (20 μM) for 2 days, and further incubated for indicated time in the presence of a proteasome inhibitor (ALLN; 20 μM). Expression of the indicated proteins was assessed by immunoblotting. (C) JEV infection attenuates cellular protein synthesis. Total protein synthesis in JEV-infected cells was determined via [^35^S]-methionine incorporation at indicated time points (left panel). The data represent the mean ± SD of three independent experiments. Significant differences were determined using Student’s *t*-test and are indicated by asterisks (*P<0.05) and double asterisks (**P<0.01). Protein synthesis of MCL1 in JEV-infected cells was determined via [^35^S]-methionine and [^35^S]-cysteine incorporation at 2 days post-infection. MCL1 proteins were immunoprecipitated with the appropriate antibody and subjected to SDS-PAGE (right panel). (D) Huh7 cells infected with JEV (MOI = 5) were incubated with QVD-OPH (20 μM) and subjected to immunoblotting at 2 days post-infection. (E) Huh7 cell lines stably expressing GFP and D2GFP were infected with JEV (MOI = 5 or 20) then incubated with QVD-OPH (20 μM) and subjected to immunoblotting at 2 days post-infection. Images (A, B, D, E) are representative of two independent experiments.

BAX/BAK activation is suppressed by pro-survival BCL2 proteins [20]. To investigate how BCLX_L_ participates in cell survival upon infection with flaviviruses, expression levels of BCLX_L_ and MCL1 were determined in flavivirus-infected cells. Interestingly, expression of MCL1 decreased upon infection with JEV, DENV and ZIKV, in contrast to the continued stable expression of other BCL2 family proteins (Fig 3A). No MCL1 protein was detected in the insoluble fraction of cell lysates (S3A Fig), suggesting that the total amount of MCL1 protein is decreased by the infection with flaviviruses.

MCL1 has been shown to be a short-lived protein that is degraded by the proteasome [32,38,39]. Northern blot analysis revealed stable expression of mRNA of MCL1 in JEV-infected cells (S3B Fig). Reduction of MCL1 protein by JEV infection was slightly restored by the treatment with a proteasome inhibitor (Ac-Leu-Leu-Nle-Aldehyde, ALLN; (20 μM), however, the expression level of MCL1 was not recoverable by the inhibition of proteasome (Fig 3B). Moreover, lysosome inhibitor treatment did not restore reduction of MCL1 protein (S3C Fig), suggesting that JEV infection suppresses host translation resulting in the reduction of MCL1 protein expression through proteasome-mediated degradation.

A previous study showed that MULE (HUWE1) is a unique BH3-containing E3 ubiquitin ligase that is associated with suppression of MCL1 expression [40]. Suppression of MCL1 expression in JEV-infected MULEKO cells was comparable to MCL1 in JEV-infected parental Huh7 cells (S3D and S3E Fig). Further, the Skp, Cullin and F-box containing complex (SCF complex) has also been shown to play a role in MCL1 degradation [41,42]. Although the overexpression of dominant negative Cullin-1 (DN-CUL1) led to an upregulation of MCL1 in non-infected cells, infection with JEV still suppressed MCL1 expression (S3F Fig). Additionally, overexpression of the BH3-only protein, NOXA, has been shown to induce suppression of MCL1 expression [32]. Suppression of MCL1 expression in JEV-infected NOXAKO cells was comparable with MCL1 in JEV-infected parental Huh7 cells (S3G and S3H Fig). Importantly, expression of MCL1K/R, which is an ubiquitination-defective mutant of MCL1, was still supressed upon JEV infection (S3I and S3J Fig). The ts20 cells are derived from Balb/c 3T3 cells and possesses a temperature-sensitive ubiquitin-activating E1 enzyme [43]. The ts20 cells were infected with JEV, then incubated for 6 h at 37°C (active E1) or 39°C (inactive E1), 2 days post-infection. Notably, the expression of p53, but not of MCL1, was restored by inhibition of E1 (S3K Fig), suggesting that ubiquitination is not involved in the suppression of MCL1 protein expression in cells infected with JEV.

To examine the effects of JEV infection on protein synthesis, JEV-infected cells were labelled with [^35^S]-methionine and [^35^S]-cysteine. The total amount of protein synthesis was significantly reduced at both 2 and 3 days post-infection with JEV (Fig 3C, left panel). Additionally, protein synthesis of MCL1 was significantly reduced by infection with JEV (Fig 3C, right panel). In addition, expression of CCND1, another short half-life protein [44,45], was also suppressed by JEV infection (Fig 3D). We hypothesized that expression of short half-life proteins was non-specifically suppressed upon infection with flaviviruses because labile proteins were constitutively degraded under inhibition of newly protein synthesis. To clarify it, we generated Huh7 cell lines stably expressing either GFP or D2GFP, a destabilized form of GFP that contains a PEST sequence [46]. Expression of D2GFP and MCL1 was decreased upon infection with JEV in a dose-dependent manner at 2 days post infection, in contrast to the stable expression of GFP (Fig 3E). These results suggest that infection with flaviviruses induces inhibition of protein translation, leading to a reduction of short half-life proteins such as MCL1.

Next, to examine the effect of viral proteins on MCL1 expression, we transfected the plasmids encoding JEV structural proteins (HA-Core and prM-E), non-structural proteins (HA-NS1, HA-NS2A, HA-NS2B, HA-NS3, HA-NS4A, HA-NS4B and HA-NS5) and control vector into Huh7 cells. Expression of viral proteins did not induce suppression of MCL1 expression (S3L Fig). These results suggest that viral propagtion is required for suppression of MCL1 expression.

### Viral propagation is required for the induction of cell death through suppression of MCL1 expression

**Fig 4:**
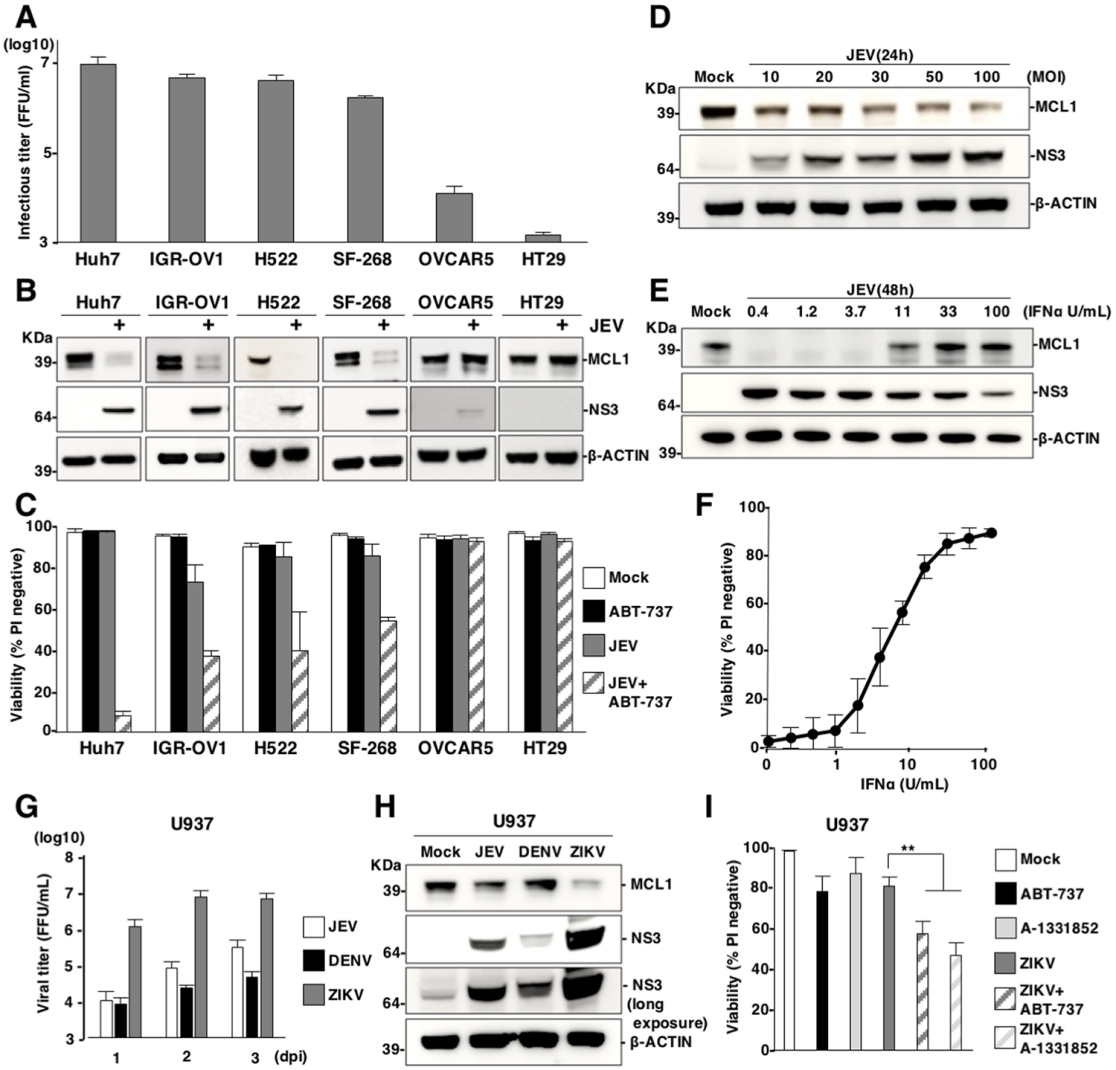
Viral propagation is required for the induction of cell death through suppression of MCL1 expression. (A) Susceptibility of human cell lines to JEV. Human cell lines were infected with JEV (MOI = 5) and infectious titers were determined by focus-forming assay at 3 days post-infection. (B) JEV-susceptible cell lines induce suppression of MCL1 expression. upon infection. Lysates from human cell lines infected with JEV (MOI = 5) were subjected to immunoblotting at 2 days post-infection. (C) Treatment with ABT-737 induces cell death in the susceptible cell lines infected with JEV. Human cell lines were infected with or without JEV (MOI = 5) in the absence or presence of ABT-737 (1 μM) and cell viability was assessed at 3 days post-infection. (D) Dose-dependent suppression of MCL1 expression by JEV infection. Huh7 cells infected with JEV, at an MOI of 10 to 100, were incubated with the caspase inhibitor, QVD-OPH (20 μM), for 1 day. Cell lysates were subjected to immunoblotting. (E) Interferon treatment attenuates JEV-induced suppression of MCL1 expression Huh7 cells infected with JEV (MOI = 5) were treated with interferon-α (IFNα). Cell lysates were subjected to immunoblotting at 2 days post-infection. (F) Interferon treatment attenuates JEV-induced cell death. Huh7 cells were infected with JEV (MOI = 5) and treated with ABT-737 (1 μM) and IFNα for 3 days. Cell viability was assessed at 3 days post-infection. (G, H) U937 cells were infected with JEV (MOI = 20), DENV (MOI = 20) and ZIKV (MOI = 20). (G) Infectious titers were determined by focus-forming assay at indicated time points, and (H) expression of MCL1 and NS3 was determined by immunoblotting at 2 days post-infection. (I) U937 cells infected with ZIKV (MOI = 20) were treated with/without ABT-737 (1 μM) or A-1331852 (1 μM) and cell viability was determined at 3 days post-infection. The data represent the mean ± SD of two (A, C, F, G) or three (I) independent experiments. Images are representative of two independent experiments (B, D, E). Significant differences were determined using Student’s *t*-test and are indicated with asterisks (*P<0.05) and double asterisks (**P<0.01).

To examine the relationship between the suppression of MCL1 expression and a susceptibility to JEV propagation in other human cell lines, we selected cell lines with high and low susceptibility to JEV infection: IGR-OV1, H522 and SF-268 cells (high susceptibility) and OVCAR5 and HT29 cells (low susceptibility) (Fig 4A). Expression of MCL1 was reduced in the highly susceptible cell lines upon infection with JEV; in contrast, MCL1 was stably expressed in cell lines with low susceptibility (Fig 4B). Moreover, cell lines with high susceptibility were killed by the combination of infection with JEV and treatment with the BH3 mimetic ABT-737 (1 μM) (Fig 4C) or A-1331852 (1 μM) (S4A Fig); in contrast, cell lines with low susceptibility remained viable when receiving this treatment. In addition, the suppression of MCL1 expression was enhanced upon infection with JEV (Fig 4D), but suppressed in a dose-dependent manner by treatment with IFNα (Fig 4E). Further, the decrease in cell viability induced by the combination treatment was reversed upon treatment with IFNα (Fig 4F). These data suggest that viral replication is important for suppression of MCL1 expression and suppression of MCL1 expression is independent on IFN signalling pathway. U937 cells, a human lymphoma cell line, exhibited high, moderate and low susceptibility to ZIKV, JEV and DENV, respectively (Fig 4G). Suppression of MCL1 expression was consistent with the degree of susceptibility to each virus at 2 days post infection (Fig 4H). Treatment with the BH3 mimetic ABT-737 (1 μM) or A-1331852 (1 μM) enhanced cell death in U937 cells that were also infected with ZIKV (Fig 4I), but not in cells that were also infected with JEV at 3 days post infection (S4B Fig). Taken together, these data suggest that suppression of MCL1 is required for an efficient viral propagation.

Primary targets of JEV, DENV and ZIKV include haematopoietic and skin cells; therefore, we next examined the effects of BCLX inhibition during flavivirus infection by using host cells closer to human infection. Monocyte-derived dendritic cells (MDDCs) have been shown to be highly susceptible to DENV infection [47]. Treatment with ABT737 (1 μM) significantly increased cell death in MDDCs that were also infected with DENV (S4C Fig). In addition, treatment with ABT-737 (1 μM) or A-1331852 (1 μM) significantly increased cell death in human melanoma cell lines, UACC257 and UACC62, upon infection with JEV (S4D Fig), and in mouse embryonic fibroblasts (MEFs) infected with JEV, DENV or ZIKV at 2 days post infection (S4E Fig). These results suggest that suppression of MCL1 expression and induction of cell death by the treatment with an inhibitor for BCLX are conserved in a variety of human and mouse cells upon infection with flaviviruses.

### Inhibition of BCLX_L_ suppresses viral production and innate immune responses

**Fig 5:**
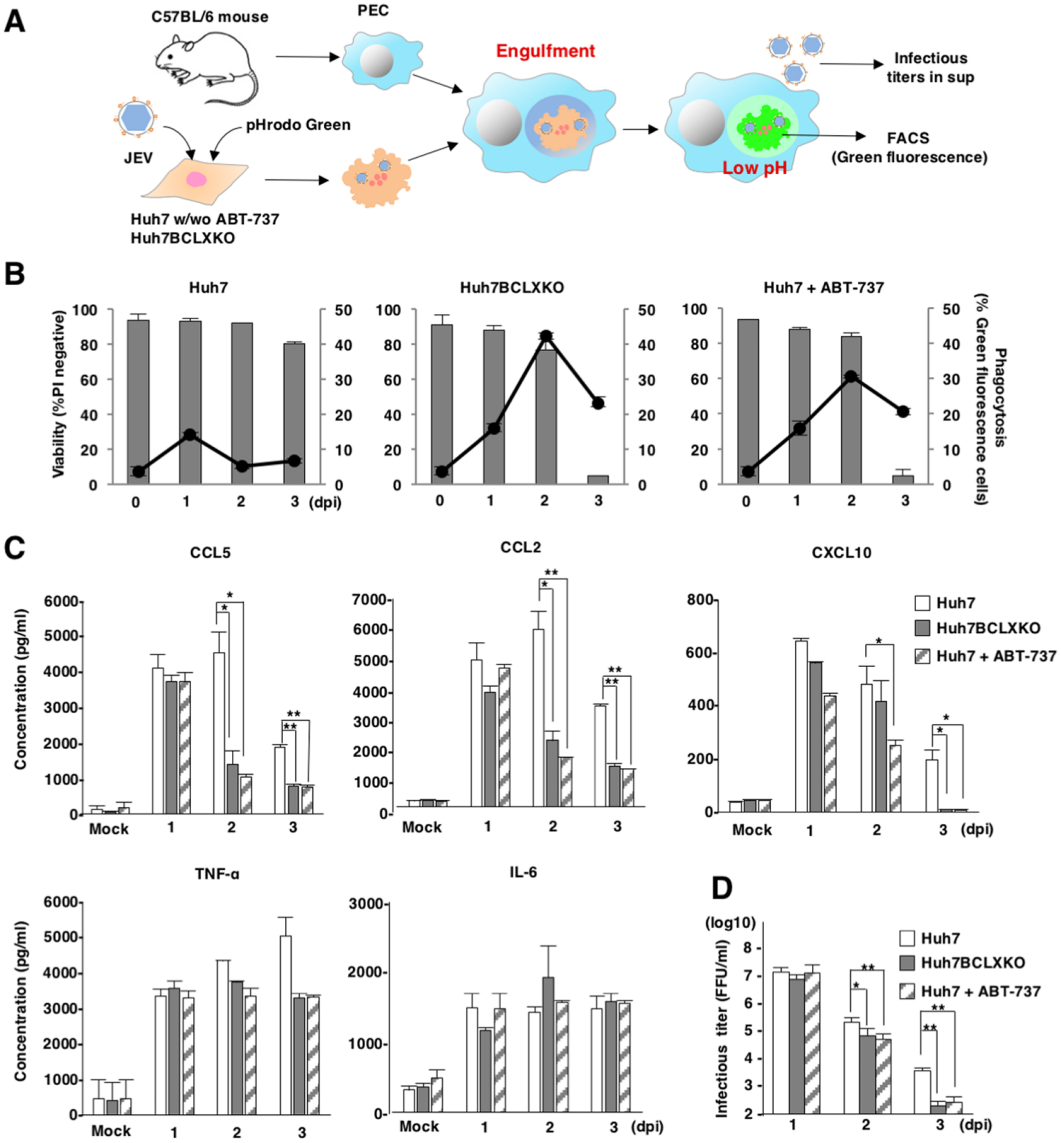
Inhibition of BCLX_L_ suppresses viral production and innate immune responses. (A) Schematic of *in vitro* phagocytosis assays. BCLXKO cells and Huh7 cells treated with/without ABT-737 (1 μM), were infected with JEV, labelled with pHrodo green at 1, 2 and 3 days post-infection and overlaid onto PECs pre-incubated overnight. Infectious titers in the supernatants and engulfment by PECs were determined by focus-forming assay and FACS analysis, respectively. (B) The percentages of engulfed cells were determined as green fluorescence by FACS analysis (line, right axis). Viability of JEV-infected BCLXKO cells and JEV-infected Huh7 cells treated with/without ABT-737 (1 μM) was assessed at indicated time points (bar, left axis). The data represent the mean ± SD of two independent experiments. (C) Suppression of viral propagation by phagocytosis of infected cells. JEV-infected BCLXKO cells and JEV-infected Huh7 cells treated with/without ABT-737 (1 μM) were overlaid onto PECs at 1, 2 and 3 days post-infection. Infectious titers in the culture supernatants were determined by focus-forming assay at 1 day after incubation. The data represent the mean ± SD of three independent experiments performed with a culture representative of each cell line. (D) Production of TNF-α, CCL5, CCL2, IL-6 and CXCL10 in the supernatants of PEC cultures (generated as in Figure 5C) was determined by ELISA. The data are representative of three independent experiments. Significant differences (C, D) were determined using Student’s *t*-test and are indicated with asterisks (*P<0.05) and double asterisks (**P<0.01).

An arthropod-borne flavivirus primarily infects dendritic cells, such as Langerhans cells, and keratinocytes in skin [3]. These infected cells are stimulated and then migrate into the lymph nodes [48], leading to propagation within the lymphatic system. We hypothesized that the induction of cell death by BCLX_L_ inhibition in flavivirus-infected cells would accelerate exposure of ‘eat-me’ signal on the surface of the cells and engulfment of infected cells by phagocytes, thus inhibiting dissemination of virus infection. To examine the involvement of phagocytosis in JEV-infected cells treated with the BH3 mimetic ABT-737, we performed an *in vitro* phagocytosis assay using peritoneal exudate cells (PECs) as model phagocytes (Fig 5A). Both of BCLXKO and ABT-737-treated Huh7 cells infected with JEV exhibited a significant increase of engulfment by PECs at 2 and 3 days post-infection, even though no cell death was observed at 2 days post-infection (Fig 5B). In contrast, no phagocytosis was observed in JEV-infected Huh7 cells (Fig 5B).

To examine whether PECs produce chemokines or cytokines upon co-culture with JEV infected cells, BCLXKO and ABT-737-treated Huh7 cells infected with JEV were overlaid onto PECs at 1, 2 and 3 days post-infection. Production of chemokines and cytokines in the supernatants was determined after 24 h of incubation. Significant amounts of chemokines and cytokines were observed in the supernatants of co-cultured PECs with JEV infected but not with mock-infected Huh7 cells (Fig 5C). Inhibition of BCLX_L_ in JEV-infected Huh7 cells suppressed the production of pro-inflammatory cytokines by PECs, including CCL5, CCL2 and CXCL10, while no significant difference in the expression of TNF-α and IL-6 was observed (Fig 5C).

To examine whether infectious particles are released from the co-culture system, the supernatants were also collected and infectious titers were determined after 24 h of co-culture. Infectious titers were significantly reduced in the supernatants of PECs that were co-cultured with either BCLXKO or ABT-737-treated Huh7 cells, compared with PECs that were co-cultured with parental Huh7 cells at 2 and 3 days post-infection with JEV (Fig 5D). Additionally, we confirmed that JEV could propagate in PECs (S5 Fig). These results suggest that the inhibition of BCLX_L_ in viral-infected cells accelerates efficient engulfment by phagocytes, thereby suppressing the production of chemokines and infectious particles.

### Suppression of BCLX_L_ confers prolonged survival rate after subcutaneous challenge with JEV

**Fig 6:**
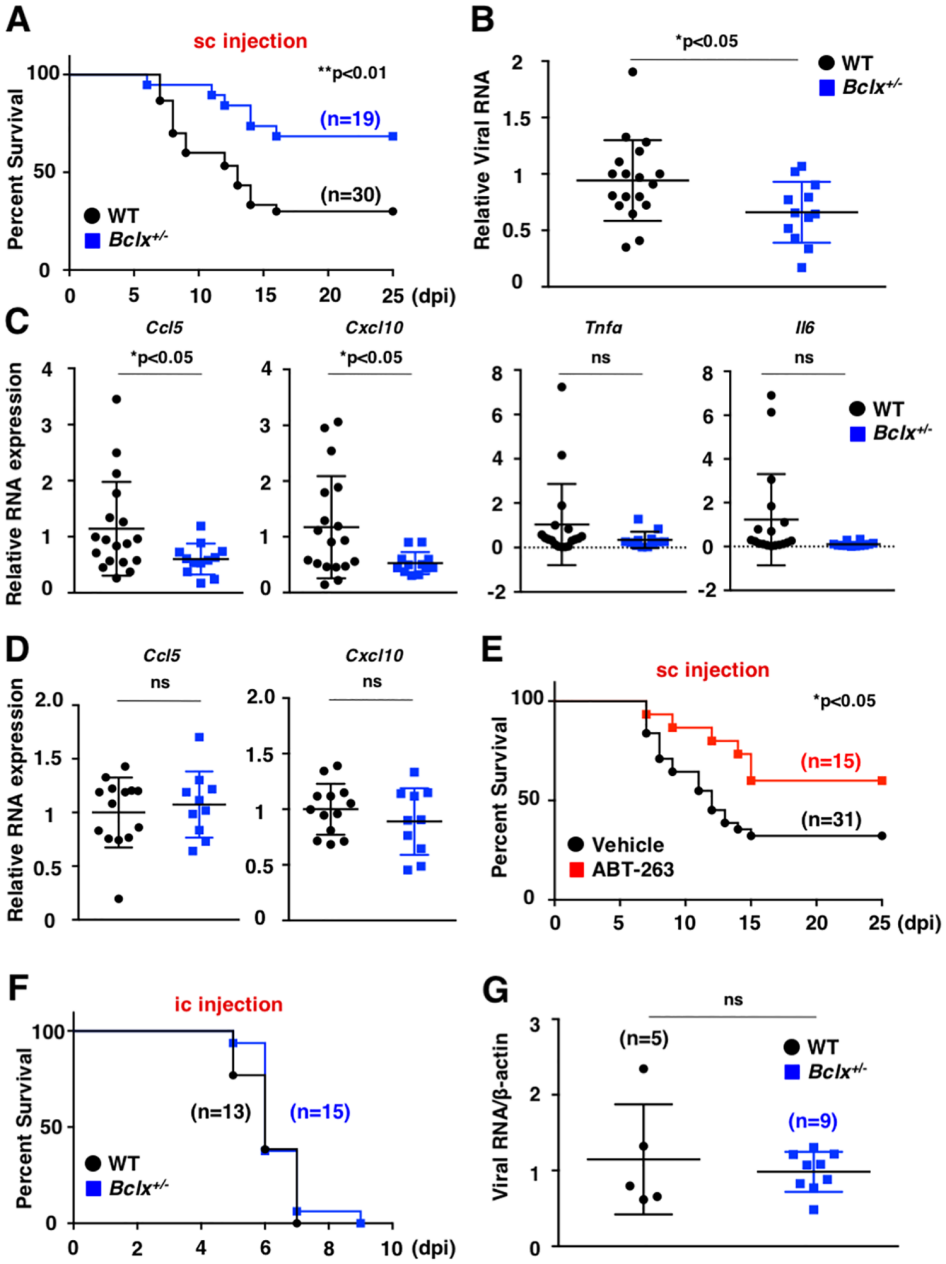
Suppression of BCLX_L_ confers higher survival rate after subcutaneous challenge with JEV. (A) Wild-type (n = 30) and *Bclx*^*+/-*^ (n = 19) mice were subcutaneously challenged with JEV (4 x 10^6^ FFU). Survival was monitored for 25 days. (B, C) Wild-type (n = 9) and *Bclx*^*+/-*^ mice (n = 6) mice were subcutaneously challenged with JEV (4 x 10^6^ FFU) and footpads were collected at 5 days post-infection. Total JEV RNA (B), and mRNA levels of *Ccl5*, *Cxcl10*, *Tnfα* and *Il6* (C) in the footpads were determined by qPCR. (D) Wild-type (n = 7) and *Bclx*^*+/-*^ mice (n = 5) were subcutaneously injected with Poly(I:C) (10 μg) suspended in 50 μL PBS and footpads were collected at 12 h post-inoculation. Expression of *Ccl5* and *Cxcl10* in the footpads of wild-type and *Bclx*^*+/-*^ mice was determined by qPCR. (E) C57BL/6 mice that were subcutaneously infected with JEV (4 x 10^6^ FFU) were orally treated with 50mg kg^-1^ of the BH3 mimetic ABT-263 (n = 15) or vehicle (n = 31) at 2 days post-infection. Survival was monitored for 25 days. (F, G) Wild-type (n = 13) and *Bclx*^*+/-*^ mice (n = 15) were intracerebrally infected with JEV (1 x 10^5^ FFU). Survivals was monitored for 10 days (F) and viral RNA was determined by qPCR (G). The relative expression (B, C, D) in *Bclx*^*+/-*^ mice was represented as the ratio of each value per leg to the corresponding value of wild-type mice using the standard curve method. The data represent each value and mean ± SD. Significant differences (B, C) were determined using Student’s *t*-test and are indicated by asterisks (*P<0.05) and double asterisks (**P<0.01). Significant differences (A, F) were determined using the log-rank test and are indicated by asterisks (*P<0.05) and double asterisks (**P<0.01).

To further examine the biological significance of BCLX_L_ inhibition in virus-infected cells, mice that were heterozygotic for *Bclx* were generated using a CRISPR/Cas9 system (S6A Fig), because homozygous mice died around E13 due to massive cell death of immature hemopoietic cells and neurons [49]. We obtained F1 mice and generated *Bclx*^*+/-*^ mice (F2) by a cross with wild-type mice. We established three *Bclx*^*+/-*^ lines from separate lines of F1 mice (No. 32, No. 40 and No. 44, S6A Fig). *Bclx*^*+/-*^ mice expressed less BCLX protein in the spleen compared to wild type mice (S6B Fig). We injected lethal amounts of JEV into the footpad of each mouse (subcutaneous challenge). *Bclx*^*+/-*^ mice exhibited higher survival rates against footpad challenge with JEV, compared with wild-type mice (Fig 6A). Expression of viral RNA, as well as *Ccl5* and *Cxcl10*, but not *Tnfα*, *Il6 Ccl2*, *Ifna and Ifnb*, was significantly decreased in the footpads of *Bclx*^*+/-*^ mice at 5 days post-infection (Fig 6B and 6C and S6C Fig). In contrast, the expression of *Ccl5*, *Cxcl10*, *Tnfα* and *Il6* was comparable in wild-type and *Bclx*^*+/-*^ mice upon stimulation with Poly(I:C) (Fig 6D and S6D Fig). In addition, oral administration of a clinically relevant dose of ABT-263 (50mg kg^-1^), an analogue of the BH3 mimetic ABT-737, conferred prolonged survival against subcutaneous challenge of JEV (Fig 6E). In contrast, *Bclx*^*+/-*^ mice exhibited similar survival rates and viral RNA production upon intracerebral challenge of JEV with wild-type mice (Fig 6F and 6G), suggesting that the suppression of virus growth in peripheral tissues participates in the delay of death and higher percentage of survival in *Bclx*^*+/-*^ mice against JEV challenge. These results suggest that viral propagation within flavivirus-infected cells can be inhibited by the suppression of BCLX_L_ and subsequent induction of apoptosis.

## Discussion

In this study, we showed that flavivirus infection suppresses expression of labile proteins such as MCL1 without observable cytotoxicity. Clear cell death, through activation of caspase 3/7, occurred only when BCLX_L_ was inhibited. Moreover, we showed that treatment with BCLX_L_ inhibitor (A-1331852) or deficiency of the *BCLX*_*L*_ gene induces cell death upon infection with flaviviruses in several cancer cell lines and primary MEFs. Previous report showed that BCLX_L_ and MCL1 predominantly inhibit activation of BAK [50], suggesting that a mechanism to inhibit BCLX_L_ may induce BAK-dependent apoptosis in flavivirus-infected cells, therefore inhibition of BCLX alone could enhanced cell death in virus-infected cells. In addition, viral infection decreased MCL1 expression in a variety of human and murine cell lines. Although the magnitude of suppression of MCL1 expression varied depending on cell line, our data suggest that suppression of MCL1 expression upon propagation of flaviviruses is a common feature. *Legionella* infection also induces suppression of MCL1 expression [51]. Although the molecular mechanisms of MCL1 protein expression in cells infected with *Legionella* remain unclear, the suppression of MCL1 through shut off of host translation might be part of the cellular innate response against infection with intracellular pathogens.

MCL1 expression is strictly controlled by transcriptional and post-transcriptional mechanisms [52]. In this study, we showed that flavivirus infection suppresses protein synthesis, thereby reducing the quantity of proteins with short half-lives, such as MCL1. In addition, recent reports have shown that infection with flaviviruses represses translation of host proteins, but does not repress viral proteins, which are independent of PKR and eIF2α [53]. Further, infection with mammalian orthoreovirus has been shown to inhibit host translation by compartmentalization of translation machinery within the viral replication complex [54]. Protein translation is regulated by ribosomal proteins [55]. DENV NS1 protein specifically binds to the ribosomal protein RPL18 to sequester in perinuclear region and to regulate viral translation and replication [56]. RPLP1 and RPLP2 identified by genome-wide RNAi screening were host factors to contribute DENV and yellow fever virus (YFV) infection [57]. We previously reported that heterogeneous nuclear ribonucleoprotein A2/B1 (HNRNPA2B1) interact with JEV core protein and facilitate JEV replication through the translocation of core protein from nucleus to cytoplasm [58]. In addition, infection with HCV has been shown to induce accumulation of adenosine 5′-triphosphate in the viral replication complex [59]. Because expression of JEV proteins exhibited no effect on host translation (S3L Fig), viral propagation might induce shut-off of host translation by unknown mechanism rather than PKR and eIF2α. On the other hand, viruses counteract host responses through the rearrangement of membrane structures to restrict the components of translation machinery into the viral replication complex.

Our data indicate that inhibition of BCLX_L_ in viral-infected cells activates phagocytosis by macrophages, leading to the suppression of infectious particle production and suggest that inhibition of BCLX_L_ function as an anti-viral effect. Although the survival of mice that were intracerebrally inoculated with JEV was not significantly different between wild-type and *Bclx*^*+/-*^ mice, *Bclx*^*+/-*^ mice showed higher survival rate against subcutaneous challenge by JEV, compared with their wild-type counterparts. Our data suggest that phagocytosis plays an important role in removing apoptotic.

JEV-infected cells after BCLX inhibition. Microglial cells, a resident subset of macrophages in the brain, and hematogenous macrophages play a role in phagocytosis in the brain and peripheral tissues, respectively. These two-types of macrophage have phagocytotic activity but have some different functions [60]. The functional difference of the microglia and hematogenous macrophages might be involved in the phagocytosis-mediated removals of virus-infected cells by BCLX inhibition in intracerebral or subcutaneous infection. Collectively, these data suggest that apoptosis induced by inhibition of BCLX_L_ attenuates viral virulence in peripheral tissues.

The production of cytokines and chemokines during viral infection is strongly associated with viral pathogenicity [61], especially, deficiency of several chemokine receptors such as CCR5 and CXCR3 promotes severity of pathogenicity against WNV infection [62]. We showed that the inhibition of BCLX_L_ specifically induces apoptosis and impaired production of CCL5 and CXCL10, which are ligands for CCR5 and CXCR3, respectively, during *in vitro* and *in vivo* viral infections. Impairment of these chemokines may contribute attenuation of pathogenicity by inhibition of BCLX_L_. In addition, administration of the BH3 mimetic, ABT-263, into wild-type mice increased survival rate after subcutaneous JEV challenge. We also showed that production of viral RNA and chemokines was impaired in Bclx^+/-^ mice (Fig 6B and 6C). In addition, treatment of wild type and Bclx^+/-^ mice with polyIC exhibited no effect on chemokine induction (Fig 6D). These data suggest that enhanced apoptosis in *Bclx*^*+/-*^ and ABT-263-treated mice upon infection with JEV promotes engulfment by phagocytes, which leads to reduces viral production and the number of leukocytes migrating to the infection site due to lower production of chemokines.

Treatment with inhibitors of BCLX such as ABT-263 was demonstrated to cause thrombocytopenia due to their neutralization of BCLXL in circulating platelets without bone marrow toxicity [30,63,64]. It was also demonstrated that thrombocytopenia induced by ABT-263 is dose-dependent and reversible effect [64], therefore it is possibility to control thrombocytopenia by appropriate terms of treatment or amounts of BCLX inhibitors [65,66]. It is also another possibility to develop the drug delivery system to virus-infected cells to avoid the unexpected side effects. Further studies are needed to use inhibitors of BCLX for antiviral therapy.

**Fig 7:**
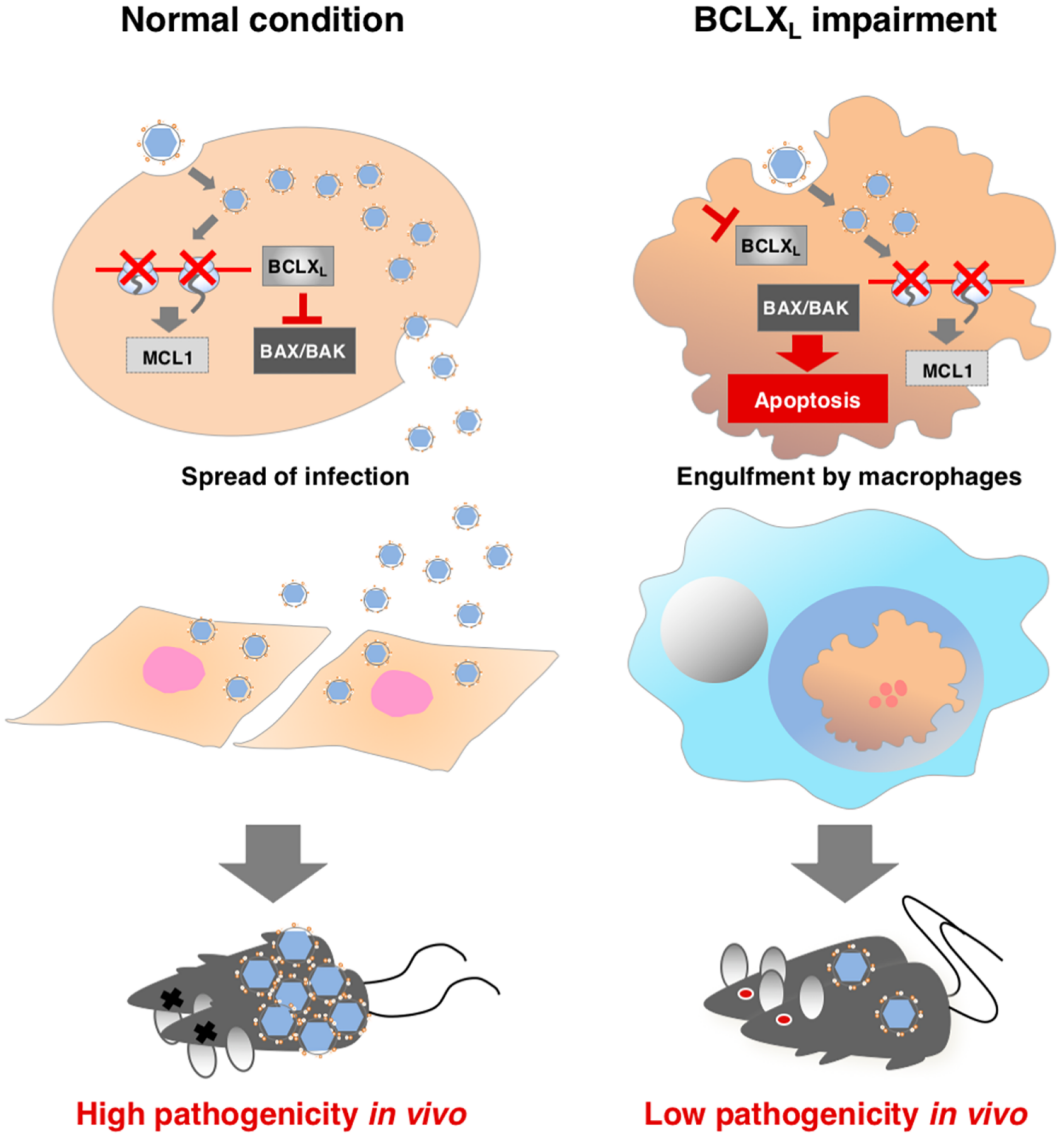
Proposal model of induction of apoptosis via BCLX_L_ inhibition. Flavivirus infection induces reduction of MCL1 protein, but BCLX_L_ delayed apoptosis in normal cells, and virus is able to spread to neighboring cells, leading to high pathogenicity. On the other hand, inhibition of BCLX_L_ accelerates apoptosis upon infection and viral dissemination is inhibited by removal of infected cells by phagocytosis.

Based on these results, we propose the model as shown in Fig 7 to explain the regulation of BCL2 family proteins upon infection with flaviviruses. In this study, we showed that infection with flaviviruses reduces expression of MCL1 but remaining BCLX_L_ delayed apoptosis in infected cells. This delay of apoptosis is crucial for viral dissemination to neighbouring cells, leading to high pathogenicity. On the other hand, impairment of BCLX_L_ accelerates apoptosis in cells infected with flavivirus and dissemination is reduced by the removal of infected cells through phagocytosis, leading to local infection and low pathogenicity *in vivo*.

In conclusion, we have demonstrated that BCLX_L_ is a critical cell survival factor during infection with flaviviruses, and that inhibition of BCLX_L_ by treatment with BH3 mimetics restricts the production of infectious particles *in vitro* and *in vivo*. We showed that expression of pro-survival MCL1 is reduced upon infection with flaviviruses. Although further studies are needed to clarify the molecular mechanisms, the translational suppression induced by viral infection is suggested to participate in the instability of unstable proteins including MCL1. *Bclx*^*+/-*^ mice exhibited impairment of pathogenicity to JEV infection, together with the enhanced viral clearance by phagocytosis of infected cells through the stimulation of apoptosis by BCLX_L_ inhibition. In addition, *Bclx*^*+/-*^ mice, and wild-type mice that received ABT-263, exhibited prolonged survival rates after JEV challenge compared with untreated wild-type mice. These data suggest that BCLX_L_ may serve as a novel antiviral target that facilitates suppression of the propagation of a broad range of the family *Flaviviridae* viruses.

## Materials and methods

### Viruses and cell lines

Huh7, 293T, U937, and Vero cells were obtained from the National Institute of Infectious Diseases and cultured in Dulbecco’s Modified Eagle’s medium (DMEM) supplemented with 10% fetal bovine serum (FBS), 100 U/ml penicillin and 100 μg/ml streptomycin. IGR-OV1, SF268, H522, OVCAR5, HT29, UACC257, UACC62 and ts20 were provided by the Walter & Eliza Hall Institute and maintained in RPMI1640 supplemented with 10% fetal bovine serum (FBS), 100 U/ml penicillin and 100 μg/ml streptomycin. The MEFs were obtained from E14.5 embryos and maintained in DMEM supplemented with 10% fetal bovine serum (FBS), 100 U/ml penicillin and 100 μg/ml streptomycin. JEV (AT31 strain), DENV (H241 strain) and ZIKV (MR766-NIID strain) were propagated in C6/36 cells. Infectious titers of JEV, DENV and ZIKV were determined by focus-forming assay. Mouse monoclonal antibodies against JEV/DENV/ZIKV NS3 (#578, #1791);[58] were used to visualize the focuses.

### Plasmids

BIM, BIM-BADBH3, BIM-NOXABH3 and BIM-4EBH3 [28] were amplified by Tks Gflex DNA Polymerase (Takara-Bio) and cloned into an FUIPW lentiviral transfer vector [67] by using an In-Fusion HD cloning Kit (Takara-Bio). The MCL1^K/R^ gene was synthesized by Thermo Fisher Scientific (GeneArt Strings), amplified and cloned into an FUIPW vector or a pCAGGS vector [68]. The cDNA of D2GFP [46] was synthesized (Integrated DNA Technologies (IDT)) and cloned into an FUIPW vector. pCAG-HA-Ubiquitin was previously described [69]. Plasmids encoding HA-tagged JEV Core and NS proteins (pCAGPM-HA-NS) were previously described [58,70]. Plasmids encoding JEV prM-E protein was previously described [71]. pcDNA3-DN-hCUL1-FLAG was obtained from Addgene (#15818). The pSPT18 was obtained from Dr. Kamitani. For the CRISPR/Cas9 system, the plasmids of pX330 (#42230) and pCAG EGxxFP (#50716) were obtained from Addgene. All sgRNAs are listed in Appendix S1 Table. The lentivirus packaging vectors, pMDLg/pRRE, pRSV-Rev and pCMV-VSV-G, were obtained from Addgene. The sequences of all plasmids used in this study were confirmed by using an ABI Prism 3130 genetic analyzer (Applied Biosystems).

### Lentivirus production

To generate lentiviruses, 293T cells were transfected with transfer vectors, pMDLg/pRRE, pRSV-Rev and pCMV-VSV-G, using polyethylenimine (PEI; MW: 25,000; Polysciences Inc.), and the culture supernatants collected at 3 days post-transfection were passed through a 0.45-mm filter. Culture supernatants containing the lentiviruses were inoculated into target cells seeded on 6-well plates using Polybrene (Sigma) and were centrifuged at 2,500 rpm for 45 min at 32 °C.

### Antibodies and reagents

The following antibodies were used: anti-JEV/DENV/ZIKV NS3 monoclonal antibody (#578, #1791, 1:1000 dilution) [58], anti-ACTIN mouse monoclonal antibody (A2228; Sigma, 1:5000 dilution), horseradish peroxidase-conjugated anti-FLAG mouse monoclonal antibody (clone M2; Sigma, 1:1000 dilution), anti-HA rat monoclonal antibody (clone 3F10; Roche, 1:1000 dilution), mouse anti-cytochrome c (BD 556433, 1:1000 dilution), rabbit polyclonal antibodies to MCL1 (sc-819; Santa Cruz, 1:1000 dilution, 600-401-394; Rockland, 1:5000 dilution), mouse monoclonal antibodies to BCLX_L_ (BD 610747, 1:1000 dilution, BD 610212, 1:1000 dilution), BAK (8F8, 1:1000 dilution), BAX (21C10-23-8-3, 1:1000 dilution), CYCLIN D1 (BD 556470, 1:1000 dilution), FKBP8 [72] (1:1000 dilution), MULE (NB100-652; Novus Biologicals, 1:1000 dilution), p62 (M162-3; MBL, 1:1000 dilution), p53 (1C12; Cell Signaling Technology, 1:1000 dilution), ABT-737, ABT-199 and A-1331852 were synthesized by Walter & Eliza Hall Institute. ABT-263 was obtained from Synkinase. S63845 was obtained from Active Biochem. ALLN (A6185) and bafilomycin A1 (B1793) were obtained from Sigma, QVD-OPH (sc-222230) was obtained from Santa Cruz Biotechnology and Digitonin (#300410) was obtained from Calbiochem, E64d (4321-v), and pepstatin A (4397-v) were obtained from Peptide Institute, Inc. human IL-4 (200–04) and GM-CSF (300–03) were obtained from peprotech. Poly(I:C) (tlrl-pic) was obtained from invivogen.

### Establishment of gene-knockout cell lines by using the CRISPR/Cas9 system

Knockout Huh7 cell lines were established by using CRISPR/Cas9 as described previously [73]. The plasmids of pX330 (#42230) and pCAG EGxxFP (#50716) were obtained from Addgene. The target sequences of each gene are summarized in S1 Table. Genomic DNAs of Huh7 were amplified and cloned into pCAG EGxxFP. Huh7 cells were transfected with pX330 and pCAG EGxxFP and incubated for 1 week. GFP-positive Huh7 cells were sorted by FACS and formed a single colony. Gene deficiency was confirmed by sequencing and Western blotting.

### Western blotting

Cell lysates were prepared by incubation in Onyx lysis buffer consisting of 20 mM Tris-HCl (pH 7.4), 135 mM NaCl, 1% Triton X-100, 1% glycerol and protease inhibitor cocktail tablets (Roche Molecular Biochemicals) for 15 min at 4°C. Cell lysates were centrifuged at 13,000 rpm for 5 min at 4°C after sonication. Protein concentrations of supernatants of cell lysates were determined by using Bio-Rad Protein Assay Dye Reagent Concentrate (Bio-Rad). The supernatants were incubated with sample buffer at 95°C for 5 min. The samples (50 μg of proteins) were resolved by SDS-PAGE (Novex, Life Technologies) and transferred onto nitrocellulose membranes (iBlot, Life Technologies). These membranes were blocked with PBS containing 5% skim milk, and then incubated with primary antibody at 4°C overnight. After washing, the membrane was reacted with HRP-conjugated secondary antibody at room temperature for 2 h. The immune complexes were visualized with Super Signal West Femto substrate (Pierce) and detected by an LAS-3000 image analyzer system (Fujifilm).

### Northern blotting

Total RNA was subjected to electrophoresis in 1.2% agarose-formaldehyde gel and morpholinepropanesulfonic acid buffer. rRNA was visualized by ethidium bromide staining, and electrophoresed RNA was transferred onto a positively charged nylon membrane (Cat No. 11 417 240 001; Roche). Full-length MCL1 cDNA was amplified and cloned into pSPT18 digested by EcoRI using an In-Fusion HD cloning kit (Clontech), an RNA probe was synthesized by using a digoxigenin (DIG) RNA labeling kit (Roche), and hybridization and detection were performed by using a DIG Northern Starter kit (Roche) according to the manufacturer’s protocols. The signals were detected by an LAS-3000 image analyzer system (Fujifilm).

### Cell death assay

Huh7 cells (2x10^4^ cells) were seeded on 12-well plates (Greiner) and incubated for 1 day. JEV, DENV or ZIKV were infected for 2 h and the culture medium was changed. At each time point, supernatants containing cells and adherent cells were collected and stained with 5 μg/mL of propidium iodide (PI, P4170; Sigma). Cell viability was determined by flow cytometry analyses (BD) and FlowJo software (FlowJo, LLC).

### Caspase activity assay

Huh7 and BAX/BAKDKO (#29 and #47) cells were infected with JEV (MOI = 5) and incubated at 37°C for 2 days. Caspase 3/7 activity was determined by using Caspase 3/7Glo (Promega) according to the manufacturer’s protocol.

### Clonogenic assay

Cells (2x10^5^) seeded on 6-well plates (Greiner) were infected with lentiviruses expressing BIM, BIM-BADBH3, BIM-NOXABH3 and BIM-4E at one day after incubation, expanded into 10 cm dishes (Greiner) at 2 days post-infection, and cultured with medium containing 1 μg/ml of puromycin for 1 week with a change of culture medium every 2 days. The remaining cells were washed with PBS, incubated with 5 mL of Giemsa’s azur-eosin-methylene blue solution (Merck Milipore: 109204) for 1 h at room temperature, and washed in water.

### Ubiquitination assay

293T cells (2x10^6^) seeded on 10 cm dishes were transfected with 1.0 μg of either pCAGGS MCL1 or pCAGGS MCL1^K/R^ and 0.05 μg of HA-ubiquitin plasmid by using PEI after 12 h of incubation. After 2 days, cells were treated with proteasome inhibitor (20 μM, ALLN) for 6 h and lysed with buffer A (50 mM Tris-HCl [pH 7.4], 150 mM NaCl, 1 mM EDTA, 1% Triton X-100, 1% sodium deoxycholate, 1% SDS) supplemented with 1 mM sodium fluoride and protease inhibitor cocktail tablets (Roche) on ice for 30 min and sonicated. Cell lysates (200 μl) were diluted with buffer A lacking SDS, incubated with anti-HA antibody (HA.11, 2 μl per sample; Covance) at 4 °C for 90 min and then further incubated with Protein G Sepharose 4B (GE Healthcare) at 4 °C for 90 min. The beads were washed five times in buffer A lacking SDS and eluted in sample buffer after incubation at 95°C for 5 min. Denatured samples were resolved by SDS-PAGE and immunoblotting.

### *In vitro* cytochrome c release assay

Huh7 and BAX/BAKDKO (#47) cells seeded on 10 cm dishes were infected with JEV (MOI = 5) and treated with ABT-737 (1μM) for 1 h at 37°C. Cells (1.5 x10^6^) were pelleted at 2 days post-infection, resuspended in 100 μl of permeabilization buffer consisting of 250 mM sucrose, 1 mM EDTA, 1 mM EGTA, 5 mM MgCl2, 100 mM KCl, 20 mM Hepes, and 0.025% of digitomin (#300410; Calbiochem) supplemented with protease inhibitor cocktail tablets, and incubated on ice for 5 min. Cell lysates were centrifuged by 13,000 rpm at 4°C for 5 min and the supernatants were collected as a soluble fraction. Pellets were resuspended with Onyx lysis buffer and then incubated on ice for 30 min, and the supernatants were collected as a pellet fraction. Both lysates were incubated with sample buffer at 95°C for 5 min, and denatured samples were resolved by SDS-PAGE and immunoblotting.

### Plaque-formation assay

Huh7 and BCLXKO Huh7 cells seeded onto 12-well or 24-well plates were inoculated with serially diluted virus and incubated for 2 h. The supernatants were removed and culture medium containing 1% methylcellulose was overlaid onto the cells. After 2 days of incubation, cells were fixed with 4% paraformaldehyde and visualized by staining with methylene blue (Sigma) or Giemsa’s azur-eosin-methylene blue solution (Merck Milipore: 109204) for 1 h at room temperature, and washed in water.

### [^35^S] Methionine-labeling of proteins

Huh7 cells or JEV-infected cells were seeded on 12-well plates and washed twice with DMEM (deficient in methionine and cysteine; Gibco 21013024), then incubated with [^35^S] methionine and [^35^S] cysteine (EasyTag Express^35^S Protein labeling mix, Perkin Elmer) in DMEM without methionine and supplemented with 10% dialyzed FBS, 100 U/ml penicillin and 100 μg/ml streptomycin for 15 min at 1, 2 and 3 days post-infection. Labeled cells were washed three times with cold PBS, then lysed with 200 μl/well radioimmunoprecipitation (RIPA) buffer, and the cell lysates were centrifuged at 13,000 rpm for 10 min at 4°C. Protein concentrations were determined by using Bio-Rad Protein Assay Dye Reagent Concentrate (Bio-Rad). Clear-sol (900 μl; Nakalai Tesque) was mixed with each supernatant (100 μl), and measured with a liquid scintillation counter (LS6500; Beckman Coulter). For detection of synthesis of MCL1 protein, Huh7 cells were seeded on 10 cm dishes, infected with JEV (MOI = 5) and incubated for 2days. These cells were washed twice with DMEM (deficient in methionine and cysteine; Gibco 21013024), then incubated with [^35^S] methionine and [^35^S] cysteine (EasyTag Express^35^S Protein labeling mix, Perkin Elmer) in DMEM without methionine and supplemented with 10% dialyzed FBS, 100 U/ml penicillin and 100 μg/ml streptomycin for 15 min at 37°C. Cell lysates were prepared by incubation in Onyx lysis buffer for 15 min at 4°C. Cell lysates were centrifuged at 13,000 rpm for 5 min at 4°C. Protein concentrations were determined by using Bio-Rad Protein Assay Dye Reagent Concentrate (Bio-Rad). Same amounts of cell lysates were incubated with 2μL of MCL1 antibody at 4°C for 90 min and then further incubated with Protein G Sepharose 4B (GE Healthcare) at 4°C for 90 min. The beads were washed three times with Onyx lysis buffer, boiled at 95°C for 5 min, and subjected to SDS-PAGE. The ^35^S-labeled proteins were visualized by using a Fujifilm bioimaging analyzer (FLA-7000, Fujifilm).

### Quantitative real-time PCR (qRT-PCR)

RNA was extracted by using ISOGEN II (Nippon Gene), and cDNAs were synthesized from the total RNA using a High Capacity RNA-to-cDNA kit (Applied Biosystems) according to the manufacturer’s instructions. JEV RNA were quantified using a TaqMan RNA-to-Ct 1-Step Kit and the ViiA7 real-time PCR system (Life Technologies). The primers of JEV were 5′-GGGTCAAAGTCATTTCTGGTCCATA’ and 5′-TCCACGCTGCTCGAA’. The following probe were used: for JEV, 50-6-FAM/ATGACCTCG/ZEN/CTCTCCC/-30IABkFQ;. mRNAs of Il6, Tnfα, Ccl5, Cxcl10, Ccl2, Ifna, Ifnb and β-actin were used for quantification of mRNA expression by using a Power SYBR green RNA-to-Ct 1-Step kit and theViiA7 real-time PCR system (Life Technologies). The following primers were used: for Il6 5′-CCACTTCACAAGTCGGAGGCTTA-3′ and 5′-GCAAGTGCATCATCGTTGTTCATAC-3′ Tnfα 5′-CAGGAGGGAGAACAGAAACTCCA-3′ and 5′-CCTGGTTGGCTGCTTGCTT-3′, Ccl5 5′-AGATCTCTGCAGCTGCCCTCA-3′ and 5′-GGAGCACTTGCTGCTGGTGTAG-3′, Cxcl10 5′-ACACCAGCCTGGCTTCCATC-3′ and 5′-TTGGAGCTGGAGCTGCTTATAGTTG-3′, Ccl2 (5′-GCATCCACGTGTTGGCTCA-3′ and 5′-CTCCAGCCTACTCATTGGGATCA-3′, Ifna1 5′-AGCCTTGACACTCCTGGTACAAATG-3′ and 5′-TGGGTCAGCTCACTCAGGACA-3′ Ifnb1 5′-ACACCAGCCTGGCTTCCATC-3′ and 5′-TTGGAGCTGGAGCTGCTTATAGTTG-3′, β-actin 5′-TTGCTGACAGGATGCAGAAG-3′ and 5′-GTACTTGCGCTCAGGAGGAG- 3′.

### Human monocyte-derived DCs (MDDCs)

Human monocyte-derived DCs were generated from healthy human blood donors using Lymphocyte Separation Solution (nacalai tesque). CD14^+^ cells were isolated after Ficoll-Hypaque gradient centrifugation using MACS CD14^+^ magnetic beads with FcR Blocking Reagent (Miltenyi Biotech) as manufacturer’s instructions. These human monocytes (5x10^5^ cells) were seeded on 24 well plates and differentiated into naive DCs by culturing for 4 days in differentiated medium, Iscove’s Modified Dulbecco’s Media containing 10% fetal bovine serum (FBS), 100 U/ml penicillin, 100 μg/ml streptomycin 10 μg/ml human IL-4 (PeproTech) and 50 μg/ml human granulocyte-macrophage colony-stimulating factor (GM-CSF) (PeproTech). MDDCs (4 x 10^5^ cells) were infected with DENV (MOI = 3) for 60 min at 37°C. Cells were then pelleted at 2,000 rpm for 5 min and resuspended in 1000 μl of differentiated medium.

### Generation of *Bclx*^*+/-*^ mice

*Bclx*^*+/-*^ mice were generated as previously described [74] using a BDF1 genetic background. The pX330 containing an sgRNA against the mouse *Bclx* gene (S1 Table) was injected into mouse zygotes and transplanted into pseudopregnant female mice. The obtained mice (F1) were crossed with wild type mice and F2 mice were analyzed by sequencing. We obtained three *Bclx*^*+/-*^ lines from different F1 mice and kept them by crossing with wild type mice.

### Phagocytosis assay

Peritoneal exudate cells (PECs) were isolated from C57BL/6 (Japan SLC, Inc.) mice intraperitoneally injected with 2 ml of 3% thioglycolate solution by rinsing the peritoneal cavity with 5 ml of PBS. Cells were pelleted by 1,500 rpm at 4 °C for 5 min and suspended in RPMI supplemented with 10% FBS, 100 U/ml penicillin and 100 μg/ml streptomycin, and cultured on 10 cm culture dishes. After 2 h of incubation, adherent cells were collected and seeded in culture medium containing 10 ng/ml human IL-4 (PeproTech), then incubated overnight at 37 °C. Huh7 and BCLXKOHuh7 cells infected with JEV were incubated with pHrodo green (10 μM; Life Technologies) at 37 °C for 30 min and washed twice with culture medium. Labeled-cells were overlaid onto PECs and incubated at 37 °C for 45 min. PECs were washed with culture medium three times to remove non-adherent cells. The amounts of engulfed PECs were determined by FACS (Becton Dickinson). Labeled cells overlaid on PEC were incubated at 37 °C for 2 h and replaced with fresh medium. Infectious titers and cytokine production in the supernatants were determined after 24 h of incubation.

### ELISA

The productions of mouse IL-6 (KMC0061, Novex), TNF-α (KMC3010, Novex), CCL5 (NMR00, R&D Systems), CCL2 (MJE00, R&D Systems) and CXCL10 (BMS6018, Thermo Fisher Scientific) were quantified by ELISA according to the manufacturer’s protocol.

### *In vivo* experiments

Three-week-old mice were anaesthetized by intraperitoneal administration of 0.75 mg kg^-1^ medetomidine (Meiji Seika), 4 mg kg^-1^ midazolam (Sandoz) and 5 mg kg^-1^ butorphanol tartrate (Meiji Seika). JEV (2x10^6^ FFU/50 μl) was injected subcutaneously into both rear footpads (4x10^6^ FFU in total). JEV (1x10^5^ FFU) was also intracerebrally injected. The survival of the mice was monitored for 25 days. To collect the brain tissues, mice were sacrificed at the end-point and then recorded as dead.

### Dosing

Dosing of ABT-263 was performed as previously described [51,64]. Briefly, ABT-263 was formulated in 10% ethanol, 30% polyethylene glycol 400, and 60% Phosal 50 PG for dosing. The mice were dosed orally once a day for 2 to 8 days with vehicle or 50mg kg^-1^ ABT-263.

### Ethics statement

All animal experiments conformed to the Guidelines for the Care and Use of Laboratory Animals, and were approved by the Institutional Committee of Laboratory Animal Experimentation (Research Institute for Microbial Diseases, Osaka University. Project number: H27-06-0). All healthy donors provided oral consent and were properly informed of the risks of the study.

### Statistical analysis

The statistical analyses were performed using GraphPad Prism (GraphPad Prism Software, Inc.). Significant differences were determined using Student’s *t*-test and are indicated with asterisks (*P<0.05) and double asterisks (**P<0.01) in each figure. Significant differences of in vivo survival data were determined using log-rank test and are indicated with asterisks (*P<0.05) and double asterisks (**P<0.01) in each figure.

## Supporting information

S1 Fig Establishment of Huh7 cell lines expressing each BIM-mutants and cell toxicity of each BH3-mimetics. (A) Establishment of BAX/BAKDKO Huh7 cell lines. Cell lysates of BAX/BAKDKO Huh7 cell lines (clones #29 and #47) were subjected to immunoblotting using antibodies against the indicated proteins. Images are representative of two independent experiments. (B) Interactions of BCL2 proteins with BH3-only proteins and BH3 mimetics. BH3-only proteins BIM and PUMA bind to all pro-survival BCL2 proteins; BAD binds to only BCL2, BCLX_L_ and BCLW; and NOXA binds only to MCL1. The binding properties of BH3 mimetics ABT-737 and ABT-263 are the same as those of BAD. In contrast, BH3 mimetics ABT-199 and A-1331852 specifically bind to BCL2 and BCLX_L_, respectively. (C) Interactions of BCL2 proteins with BIM-mutants. BIM binds to all pro-survival BCL2-like proteins (BCL2, BCLX_L_, BCLW and MCL1). BIM-BADBH3 binds only to BCL2, BCLX_L_ and BCLW. BIM-NOXABH3 binds only to MCL1. BIM-4E was produced by the replacement of four hydrophobic residues in the BH3 region of BIM with glutamate residues; the resulting mutant was incapable of binding to any BCL2 protein. “BCL” represents BCL2, BCLXL and BCLW. (D) Establishment of Huh7 cell lines stably expressing BIM-mutants. Cell lysates from Huh7 cell lines stably expressing BIM-mutants were subjected to immunoblotting using antibodies against the indicated proteins. Images are representative of two independent experiments. (E) Characterization of Huh7 cell lines stably expressing BIM-mutants. Huh7 cell lines stably expressing BIM-mutants were infected with lentiviruses expressing the indicated BIM-mutants; then, cell viability was assessed by PI staining and FACS analysis at 2 days post-infection. The data represent the mean ± SD of two independent experiments performed with a culture representative of each cell line. (F) Huh7 cells were treated with ABT-737, A-1331852 or ABT-199 at the indicated concentrations. Cell viability was assessed at 3 days post-treatment. The data represent the mean ± SD of two independent experiments. (G) Parental and BAX/BAKDKO (clone #47) Huh7 cell lines were infected with JEV (MOI = 5) and treated with ABT-737 (1 μM). Cells were collected at 2 days post-infection, permeabilized with digitonin and fractionated into pellet (P) and supernatant (S) fractions. Each fraction was subjected by immunoblotting using antibodies against the cytochrome c (Cyt c), BAK, β-ACTIN (cytosolic marker) and FKBP8 (membrane fraction marker).
(TIF)

S2 Fig Establishment of several independent cell clones of BCLXKO Huh7 cells are sensitive flavivirus infection. (A) Parental, BCLXKO and MCL1KO Huh7 cell lines were treated with/without A-1331852 (1 μM), S63845 (1 μM) or combination of A-1331852 (1 μM), S63845 (1 μM). Cell viability was assessed at 6h post-treatment. The data represent the mean ± SD of two independent experiments. (B) Establishment of BCLXKO Huh7 cell lines. Cell lysates of parental and BCLXKO Huh7cell lines (#1, #2, #26, #41, #42) were subjected to immunoblotting, using antibodies against the indicated proteins. Images are representative of two independent experiments performed with a culture representative of each cell line. (C) Deficiency of BCLX_L_ accelerates cell death upon infection with JEV. Parental and BCLXKO Huh7 cell lines (clones #1, #2, #26, #41, #42) were infected with JEV (MOI = 5) and cell viability was assessed by PI staining, and by FACS analysis, at 3 days post-infection. The data represent the mean ± SD of two independent experiments performed with a culture representative of each cell line.
(TIF)

S3 Fig Additional information on the mechanism of suppression of MCL1 expression. (A) Huh7 cells were infected with JEV (MOI = 5), incubated with a caspase inhibitor, QVD-OPH (20 μM), for 2 day. Cell lysates were separated into supernatant (S) and pellet (P) fractions by centrifugation. Fractions were subjected to SDS-PAGE and immunoblotting. (B) Quantity of MCL1 mRNA in Huh7 cells infected with JEV. Total RNA was extracted from Huh7 cells infected with JEV (MOI = 5) at the indicated time points, then subjected to Northern blotting. (C) Huh7 cells were infected with JEV (MOI = 5), incubated with QVD-OPH (20 μM) for 36 h, and then incubated for 12 h in the presence of a lysosome inhibitor, either E64d (30 μM)/pepstatin A (1.5μM; E64d & PEP) or bafilomycin (BAF; 10 nM). (D) Establishment of MULEKO Huh7 cell lines. Cell lysates of parental and MULEKO (clones #15 and #36) Huh7 cell lines were subjected to immunoblotting using antibodies against the indicated proteins. Images are representative of two independent experiments performed with a culture representative of each cell line. (E) MULE is not required for suppression of MCL1 expression in JEV-infected cells. Parental and MULEKO (clones #15 and #36) Huh7 cell lines infected with JEV (MOI = 5) were incubated with QVD-OPH (20 μM). Cells were subjected to immunoblotting using antibodies against the indicated proteins at 2 days post-infection. Images are representative of two independent experiments performed with a culture representative of each cell line. (F) Effect of Cullin1 inhibition on suppression of MCL1 expression upon JEV infection. Huh7 cells transfected with empty vector or DN-CUL1 plasmid were infected with JEV (MOI = 5) at 1 day post-transfection in the presence of QVD-OPH (20 μM). Cell lysates were subjected to immunoblotting using antibodies against the indicated proteins at 2 days post-infection. Images are representative of two independent experiments. (G) Establishment of NOXAKO Huh7 cell lines. Parental and NOXAKO (clones #19 and #21) Huh7 cell lines were infected with JEV and total RNAs were extracted at 2 days post-infection. RT-PCR was performed using the following primers: for NOXA, 5′-GTTTGTCTCCAAATCTCCTGAGTT-3′ and 5′-CGGAGATGCCTGGGAAGAAG-3′; and for GAPDH, 5′-TGTAGTTGAGGTCAATGAAGGG-3′ and 5′-ACATCGCTCAGACACCATG-3′. Images are representative of two independent experiments performed with a culture representative of each cell line. (H) NOXA is not necessary for suppression of MCL1 expression induced by JEV infection. Huh7 and NOXAKO cell lines (clones #19 and #21) infected with JEV (MOI = 5) were incubated with QVD-OPH (20 μM), then subjected to immunoblotting using antibodies against the indicated proteins at 2 days post-infection. Images are representative of two independent experiments performed with a culture representative of each cell line. (I) Wild-type and mutant MCL1 (MCL1K/R), in which all Lys residues were replaced with Arg, were co-transfected with HA-Ubiquitin into 293T cells. Cell lysates were immunoprecipitated using an anti-HA antibody and precipitates were subjected to immunoblotting as indicated. Images are representative of two independent experiments. (J) MCL1KO Huh7 cell line expressing either wild type or mutant MCL1 replaced all lysine residues to Arg (MCL1^K/R^) were infected with JEV (MOI = 20) and incubated with QVD-OPH (20 μM). Cells were subjected to immunoblotting at 2 days post-infection. (K) ts20 temperature-sensitive ubiquitin-activating E1 enzyme cells infected with JEV (MOI = 100) were incubated with QVD-OPH (20 μM). At 2 days post-infection, cells were either incubated at 37°C (active E1) or 39°C (inactive E1) for 6 h. Cell lysates were subjected to immunoblotting using antibodies against the indicated proteins. Images are representative of two independent experiments. (L) Cell lysates of Huh7 cells transfected with the expression plasmids were harvested at 2 days post-transfection and subjected to immunoblotting using antibodies against the indicated proteins. Images are representative of two independent experiments.
(TIF)

S4 Fig Suppression of MCL1 proteins and cell death by treatment with ABT-737 or A-1331852 in JEV infected skin cells. (A) Treatment with A-1331852 induces cell death in the susceptible cell lines infected with JEV. Viability of human cell lines infected with/without JEV (MOI = 5) in the absence or presence of A-1331852 (1 μM) was assessed at 3 days post-infection. Data are the mean of two independent experiments, represented as the mean ± SD. (B) U937 cells infected with JEV (MOI = 20) were treated with ABT-737 (1 μM) and cell viability was determined at 3 days post-infection. Data are the mean of four independent experiments, represented as the mean ± SD. (C) MDDCs infected with or without DENV (MOI = 3) were treated with ABT-737 (1 μM) and cell viability was determined at 3 days post-infection. (D) UACC257 and UACC62 cells infected with or without JEV (MOI = 20) were treated with ABT-737 (1 μM) or A-1331852 (1 μM) and cell viability was determined at 3 days post-infection. Data are the mean of four independent experiments, represented as the mean ± SD. (E) MEFs infected with either JEV (MOI = 20), DENV (MOI = 20) or ZIKV (MOI = 20) were treated with ABT-737 (1 μM) or A-1331852 (1 μM) and cell viability was determined at 3 days post-infection. Data are the mean of three independent experiments, represented as the mean ± SD. Significant differences were determined using Student’s *t*-test and are indicated by asterisks (*P<0.05) and double asterisks (**P<0.01).
(TIF)

S5 Fig Propagation of JEV in PECs. PECs were infected with JEV (MOI = 5) and infectious titers were determined by focus-forming assay at 1–4 days post-infection. The data represent the mean ± SD of two independent experiments.
(TIF)

S6 Fig Establishment of *Bclx*^*+/-*^ mice. (A) Schematic of the establishment of *Bclx*^*+/-*^ mice by the CRISPR/Cas9 system. BDF1-derived zygotes were injected with a px330 vector targeting the mouse *Bclx* gene and transplanted into pseudopregnant ICR mice. Newborn F1 mice were crossed with wild-type BDF1 mice and F2 mice were obtained. DNA sequences surrounding the *Bclx* gene in F2 mice were confirmed by sequencing. We obtained three *Bclx*^*+/-*^ mouse lines from separate F1 mouse lines (No. 32, No. 40, and No. 44). (B) Spleen lysates from wild-type or *Bclx*^*+/-*^ mice were subjected to immunoblotting using antibodies against the indicated proteins. (C) Wild-type (n = 9) and *Bclx*^*+/-*^ mice (n = 6) mice were subcutaneously challenged with JEV (4 x 10^6^ FFU) and footpads were collected at 5 days post-infection. mRNA levels of *Ccl2*, *Ifna* and *Ifnb* in the footpads were determined by qPCR. (D) Wild-type (n = 7) and *Bclx*^*+/-*^ mice (n = 5) were subcutaneously injected with Poly(I:C) (10 μg) suspended in 50 μL PBS and footpads were collected at 12 h post-inoculation. Expression of *Tnfα and Il6* in the footpads of wild-type and *Bclx*^*+/-*^ mice was determined by qPCR. The relative expression in *Bclx*^*+/-*^ mice was represented as the ratio of each value per leg to the corresponding value of wild-type mice using the standard curve method. The data represent each value and mean ± SD.
(TIF)

S1 Table DNA oligos for generating gene-knockout cell lines. (PDF)
