# Supplementary figures and images for: Infection with flaviviruses requires BCLXL for cell survival
Source: PLoS Pathog. 2018 Sep 27;14(9):e1007299. doi: 10.1371/journal.ppat.1007299 (PMC6177207; doi:10.1371/journal.ppat.1007299)

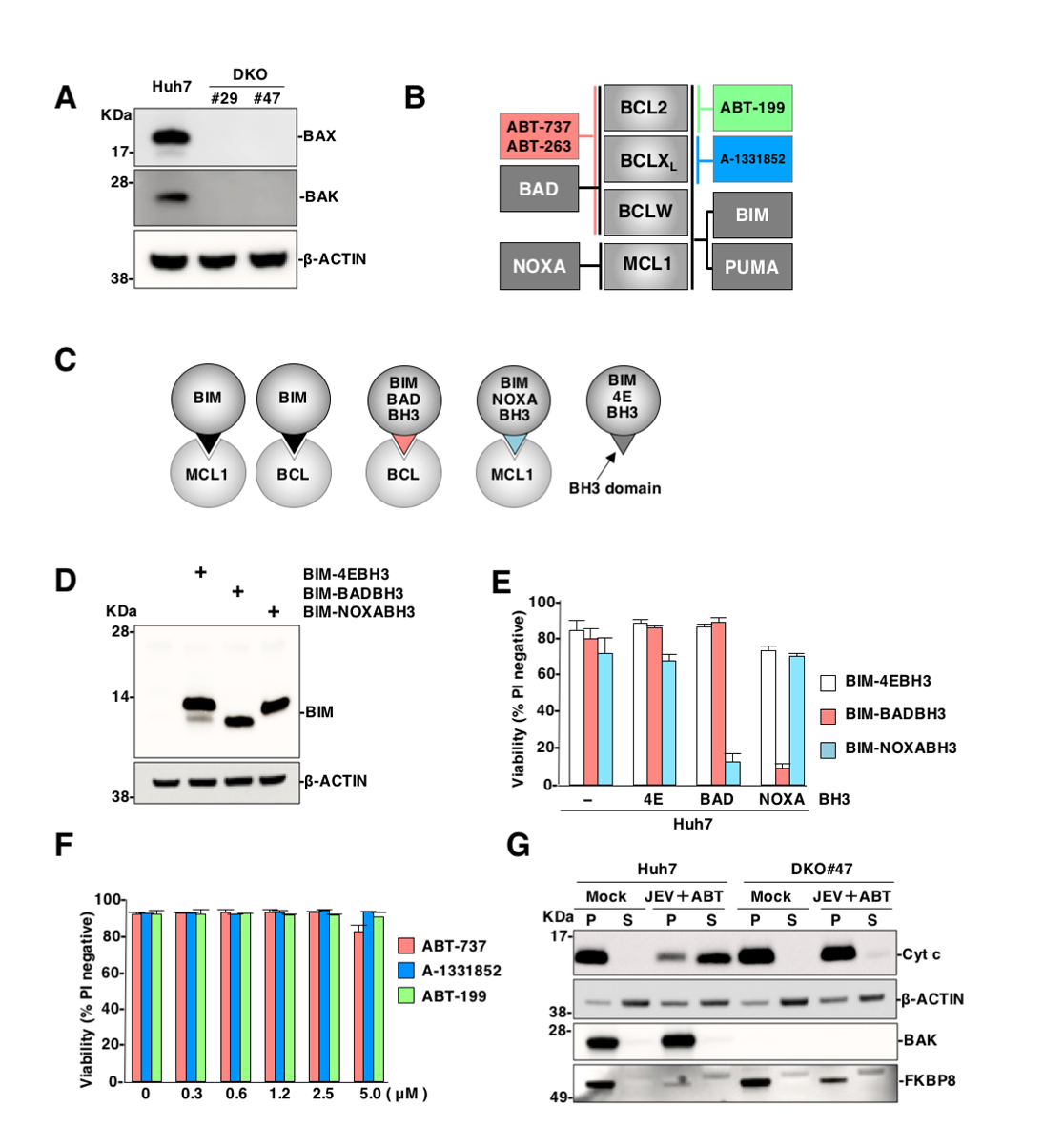

Supplement: S1 Fig — (A) Establishment of BAX/BAKDKO Huh7 cell lines. Cell lysates of BAX/BAKDKO Huh7 cell lines (clones #29 and #47) were subjected to immunoblotting using antibodies against the indicated proteins. Images are representative of two independent experiments. (B) Interactions of BCL2 proteins with BH3-only proteins and BH3 mimetics. BH3-only proteins BIM and PUMA bind to all pro-survival BCL2 proteins; BAD binds to only BCL2, BCLXL and BCLW; and NOXA binds only to MCL1. The binding properties of BH3 mimetics ABT-737 and ABT-263 are the same as those of BAD. In contrast, BH3 mimetics ABT-199 and A-1331852 specifically bind to BCL2 and BCLXL, respectively. (C) Interactions of BCL2 proteins with BIM-mutants. BIM binds to all pro-survival BCL2-like proteins (BCL2, BCLXL, BCLW and MCL1). BIM-BADBH3 binds only to BCL2, BCLXL and BCLW. BIM-NOXABH3 binds only to MCL1. BIM-4E was produced by the replacement of four hydrophobic residues in the BH3 region of BIM with glutamate residues; the resulting mutant was incapable of binding to any BCL2 protein. “BCL” represents BCL2, BCLXL and BCLW. (D) Establishment of Huh7 cell lines stably expressing BIM-mutants. Cell lysates from Huh7 cell lines stably expressing BIM-mutants were subjected to immunoblotting using antibodies against the indicated proteins. Images are representative of two independent experiments. (E) Characterization of Huh7 cell lines stably expressing BIM-mutants. Huh7 cell lines stably expressing BIM-mutants were infected with lentiviruses expressing the indicated BIM-mutants; then, cell viability was assessed by PI staining and FACS analysis at 2 days post-infection. The data represent the mean ± SD of two independent experiments performed with a culture representative of each cell line. (F) Huh7 cells were treated with ABT-737, A-1331852 or ABT-199 at the indicated concentrations. Cell viability was assessed at 3 days post-treatment. The data represent the mean ± SD of two independent experiments. (G) Parental and BAX [file ppat.1007299.s001.tif]

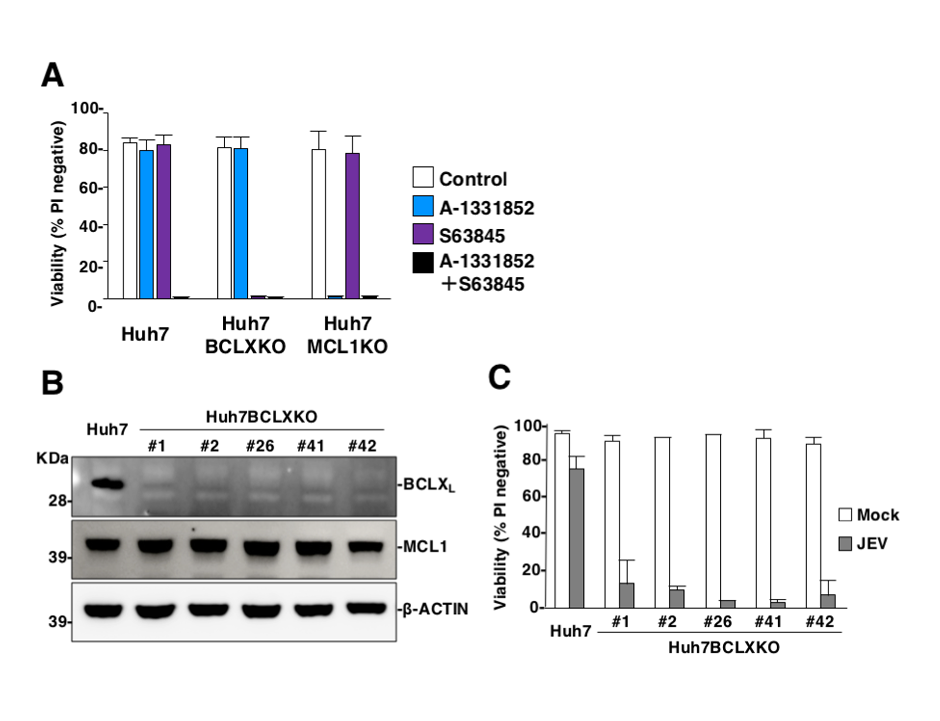

Supplement: S2 Fig — (A) Parental, BCLXKO and MCL1KO Huh7 cell lines were treated with/without A-1331852 (1 μM), S63845 (1 μM) or combination of A-1331852 (1 μM), S63845 (1 μM). Cell viability was assessed at 6h post-treatment. The data represent the mean ± SD of two independent experiments. (B) Establishment of BCLXKO Huh7 cell lines. Cell lysates of parental and BCLXKO Huh7cell lines (#1, #2, #26, #41, #42) were subjected to immunoblotting, using antibodies against the indicated proteins. Images are representative of two independent experiments performed with a culture representative of each cell line. (C) Deficiency of BCLXL accelerates cell death upon infection with JEV. Parental and BCLXKO Huh7 cell lines (clones #1, #2, #26, #41, #42) were infected with JEV (MOI = 5) and cell viability was assessed by PI staining, and by FACS analysis, at 3 days post-infection. The data represent the mean ± SD of two independent experiments performed with a culture representative of each cell line. (TIF) [file ppat.1007299.s002.tif]

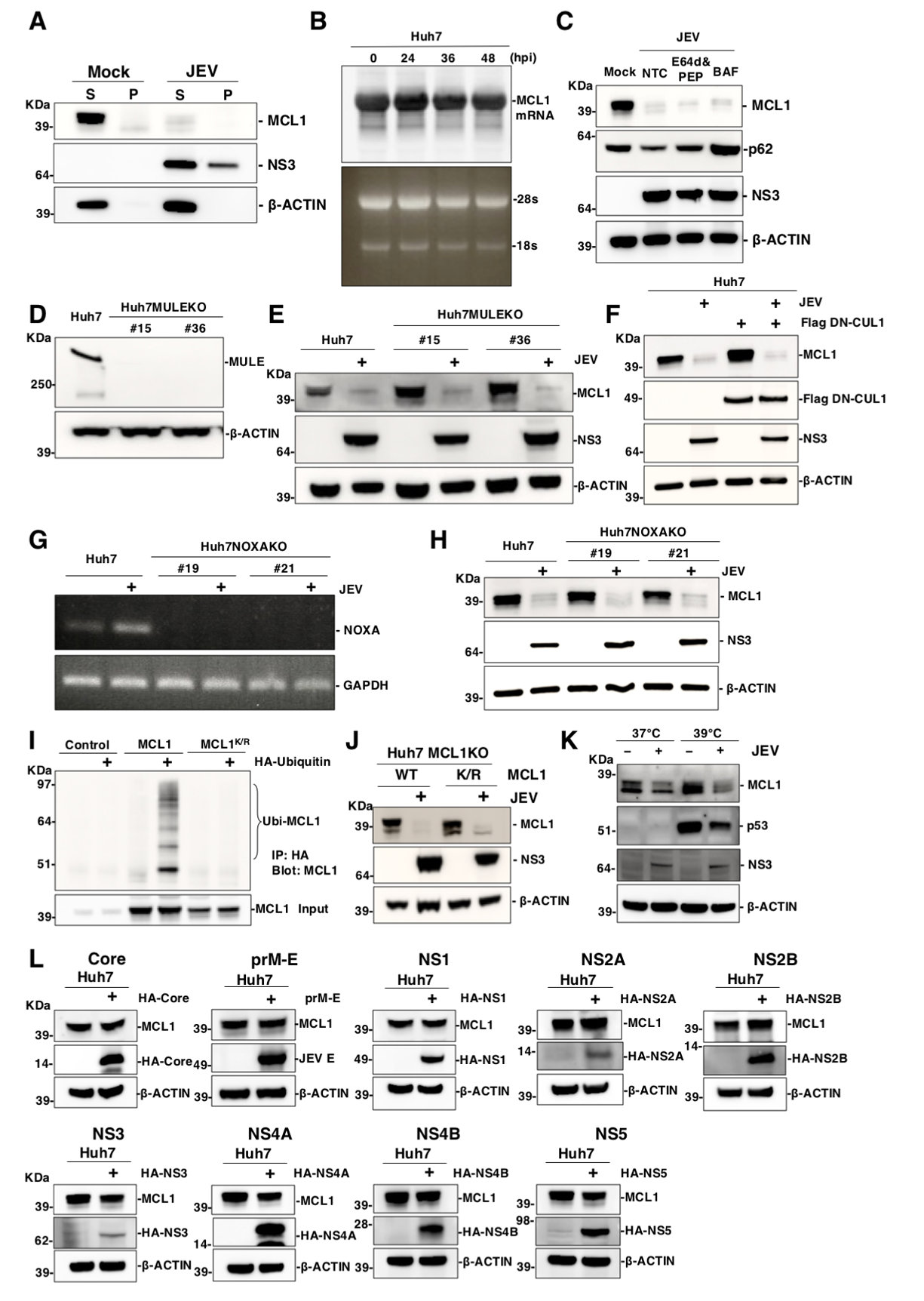

Supplement: S3 Fig — (A) Huh7 cells were infected with JEV (MOI = 5), incubated with a caspase inhibitor, QVD-OPH (20 μM), for 2 day. Cell lysates were separated into supernatant (S) and pellet (P) fractions by centrifugation. Fractions were subjected to SDS-PAGE and immunoblotting. (B) Quantity of MCL1 mRNA in Huh7 cells infected with JEV. Total RNA was extracted from Huh7 cells infected with JEV (MOI = 5) at the indicated time points, then subjected to Northern blotting. (C) Huh7 cells were infected with JEV (MOI = 5), incubated with QVD-OPH (20 μM) for 36 h, and then incubated for 12 h in the presence of a lysosome inhibitor, either E64d (30 μM)/pepstatin A (1.5μM; E64d & PEP) or bafilomycin (BAF; 10 nM). (D) Establishment of MULEKO Huh7 cell lines. Cell lysates of parental and MULEKO (clones #15 and #36) Huh7 cell lines were subjected to immunoblotting using antibodies against the indicated proteins. Images are representative of two independent experiments performed with a culture representative of each cell line. (E) MULE is not required for suppression of MCL1 expression in JEV-infected cells. Parental and MULEKO (clones #15 and #36) Huh7 cell lines infected with JEV (MOI = 5) were incubated with QVD-OPH (20 μM). Cells were subjected to immunoblotting using antibodies against the indicated proteins at 2 days post-infection. Images are representative of two independent experiments performed with a culture representative of each cell line. (F) Effect of Cullin1 inhibition on suppression of MCL1 expression upon JEV infection. Huh7 cells transfected with empty vector or DN-CUL1 plasmid were infected with JEV (MOI = 5) at 1 day post-transfection in the presence of QVD-OPH (20 μM). Cell lysates were subjected to immunoblotting using antibodies against the indicated proteins at 2 days post-infection. Images are representative of two independent experiments. (G) Establishment of NOXAKO Huh7 cell lines. Parental and NOXAKO (clones #19 and #21) Huh7 cell lines were infected with JEV and tot [file ppat.1007299.s003.tif]

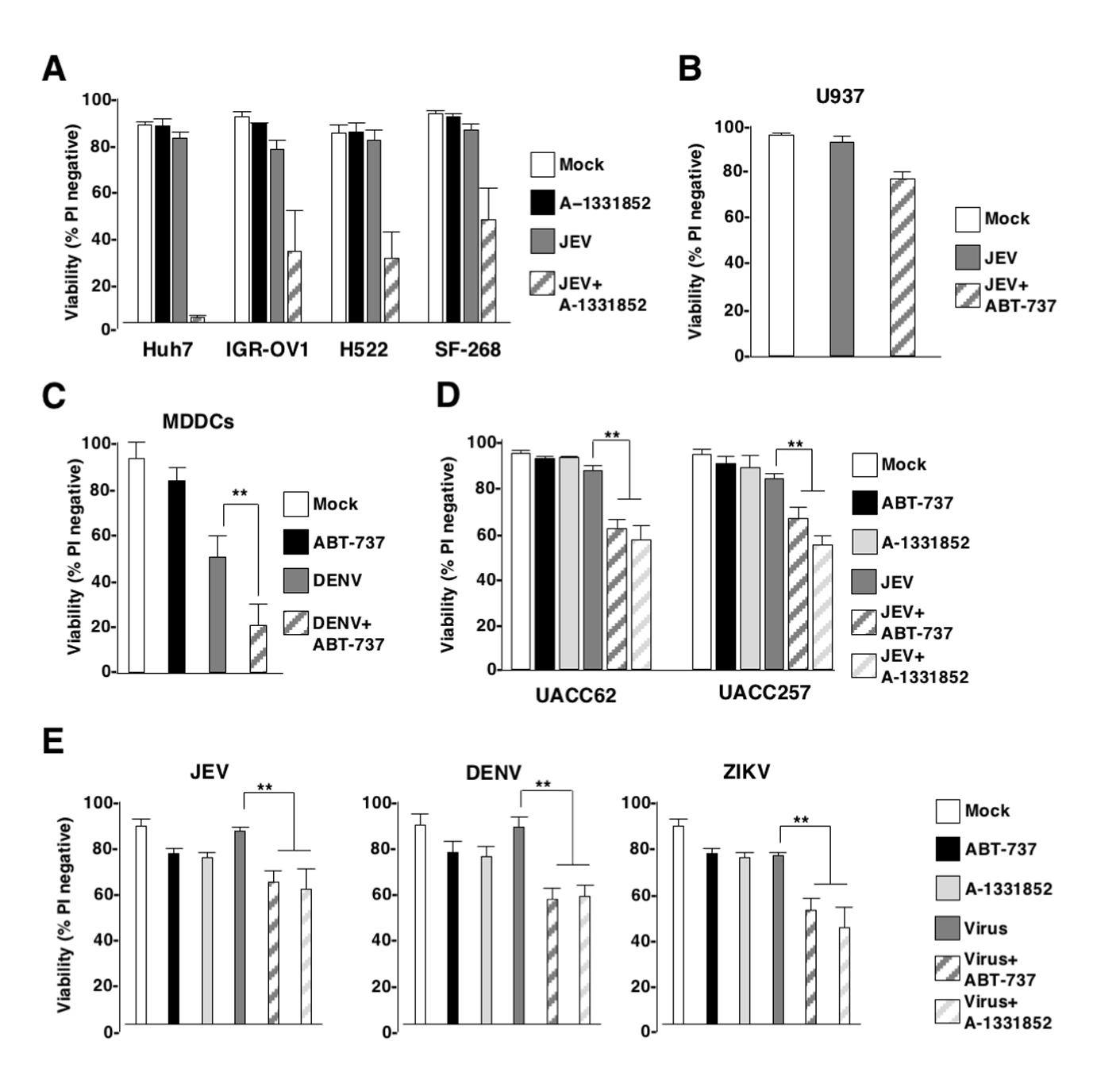

Supplement: S4 Fig — (A) Treatment with A-1331852 induces cell death in the susceptible cell lines infected with JEV. Viability of human cell lines infected with/without JEV (MOI = 5) in the absence or presence of A-1331852 (1 μM) was assessed at 3 days post-infection. Data are the mean of two independent experiments, represented as the mean ± SD. (B) U937 cells infected with JEV (MOI = 20) were treated with ABT-737 (1 μM) and cell viability was determined at 3 days post-infection. Data are the mean of four independent experiments, represented as the mean ± SD. (C) MDDCs infected with or without DENV (MOI = 3) were treated with ABT-737 (1 μM) and cell viability was determined at 3 days post-infection. (D) UACC257 and UACC62 cells infected with or without JEV (MOI = 20) were treated with ABT-737 (1 μM) or A-1331852 (1 μM) and cell viability was determined at 3 days post-infection. Data are the mean of four independent experiments, represented as the mean ± SD. (E) MEFs infected with either JEV (MOI = 20), DENV (MOI = 20) or ZIKV (MOI = 20) were treated with ABT-737 (1 μM) or A-1331852 (1 μM) and cell viability was determined at 3 days post-infection. Data are the mean of three independent experiments, represented as the mean ± SD. Significant differences were determined using Student’s t-test and are indicated by asterisks (*P<0.05) and double asterisks (**P<0.01). (TIF) [file ppat.1007299.s004.tif]

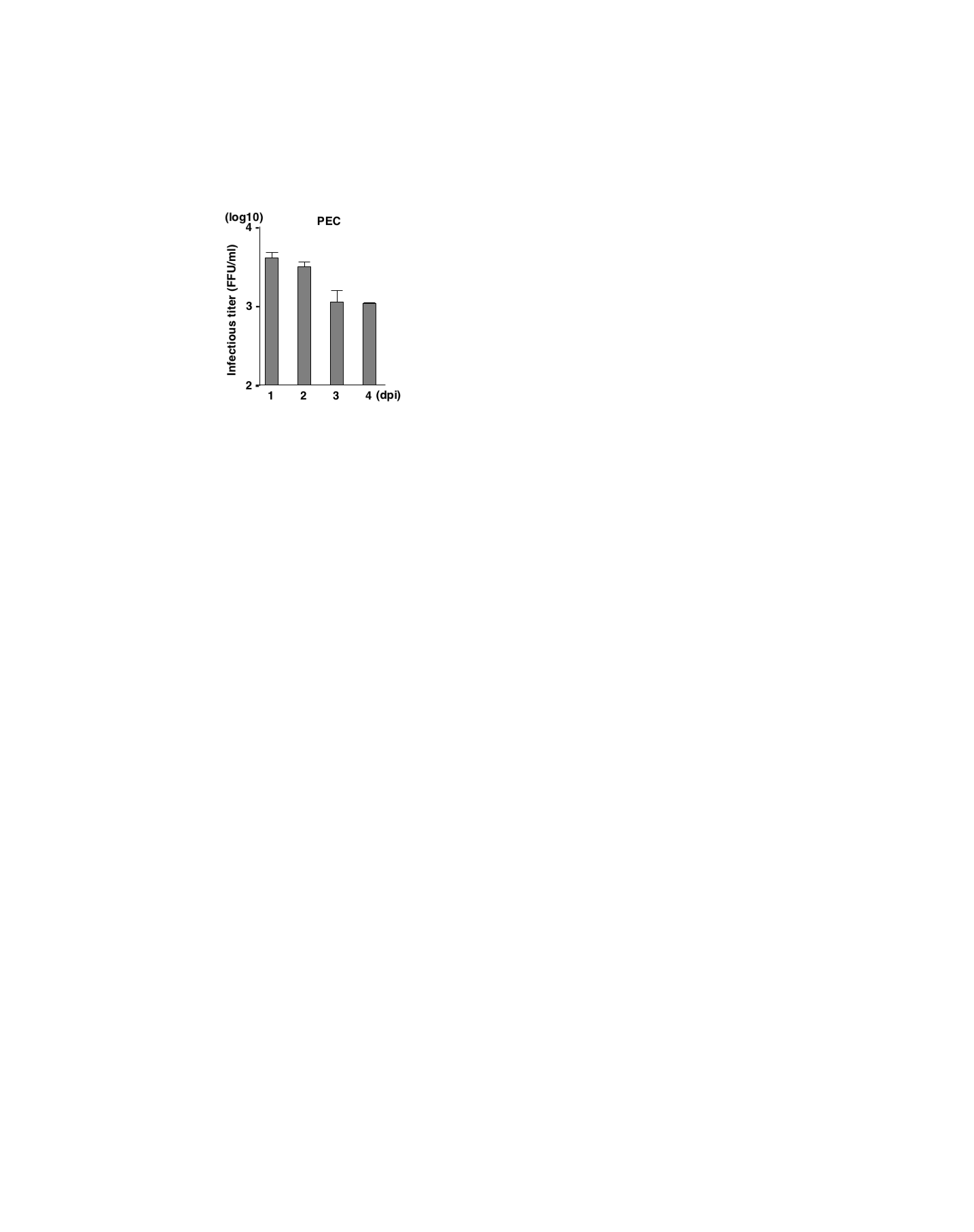

Supplement: S5 Fig — PECs were infected with JEV (MOI = 5) and infectious titers were determined by focus-forming assay at 1–4 days post-infection. The data represent the mean ± SD of two independent experiments. (TIF) [file ppat.1007299.s005.tif]

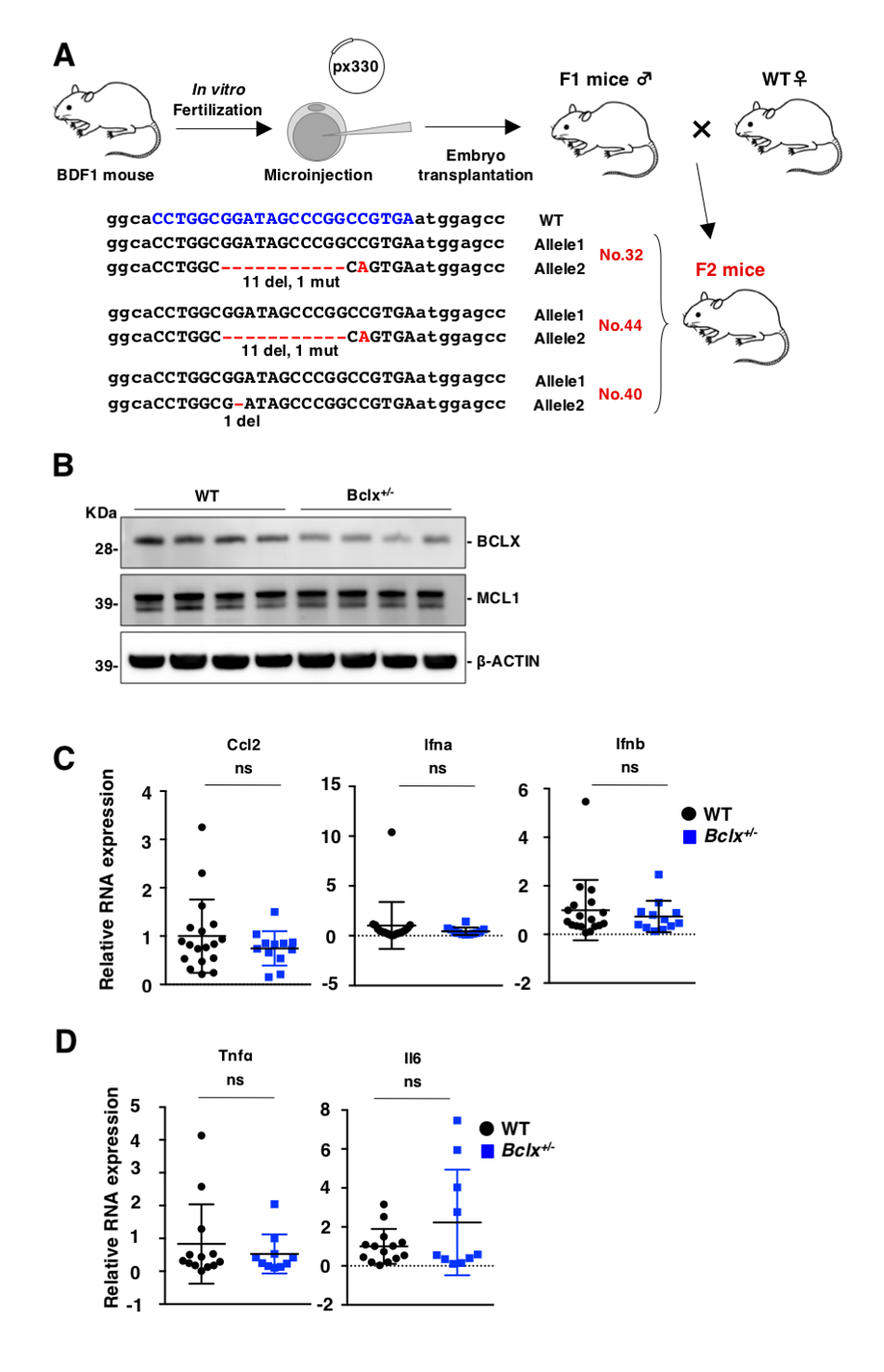

Supplement: S6 Fig — (A) Schematic of the establishment of Bclx+/- mice by the CRISPR/Cas9 system. BDF1-derived zygotes were injected with a px330 vector targeting the mouse Bclx gene and transplanted into pseudopregnant ICR mice. Newborn F1 mice were crossed with wild-type BDF1 mice and F2 mice were obtained. DNA sequences surrounding the Bclx gene in F2 mice were confirmed by sequencing. We obtained three Bclx+/- mouse lines from separate F1 mouse lines (No. 32, No. 40, and No. 44). (B) Spleen lysates from wild-type or Bclx+/- mice were subjected to immunoblotting using antibodies against the indicated proteins. (C) Wild-type (n = 9) and Bclx+/- mice (n = 6) mice were subcutaneously challenged with JEV (4 x 10^6^ FFU) and footpads were collected at 5 days post-infection. mRNA levels of Ccl2, Ifna and Ifnb in the footpads were determined by qPCR. (D) Wild-type (n = 7) and Bclx+/- mice (n = 5) were subcutaneously injected with Poly(I:C) (10 μg) suspended in 50 μL PBS and footpads were collected at 12 h post-inoculation. Expression of Tnfα and Il6 in the footpads of wild-type and Bclx+/- mice was determined by qPCR. The relative expression in Bclx+/- mice was represented as the ratio of each value per leg to the corresponding value of wild-type mice using the standard curve method. The data represent each value and mean ± SD. (TIF) [file ppat.1007299.s006.tif]
