# Supplementary material for: Infection with flaviviruses requires BCLXL for cell survival
Source: PLoS Pathog. 2018 Sep 27;14(9):e1007299. doi: 10.1371/journal.ppat.1007299 (PMC6177207; doi:10.1371/journal.ppat.1007299)
Supplement: S1 Table — (PDF) [file ppat.1007299.s007.pdf]

Supporting Information

S1 Table. DNA oligos for generating gene-knockout cell lines.

	Target sequence for Cas9<br>Forward primer for cloning into pCAG EGxxFP and sequencing<br>Reverse primer for cloning into pCAG EGxxFP and sequencing
Human BCLX	5’- GAGACCCCCAGTGCCATCAATGG -3’<br>5’- CAACCACTGAGGATCCCGGACTCAGACCTTCATAAGAGC -3’<br>5’- TGCCGATATCGAATTCCAAGGGTAGCCAGGATGAAAGA -3’
Human MCL1	5’- GGACTCAACCTCTACTGTGGGGG -3’<br>5’- CAACCACTGAGGATCCTCACAAATCAGGTCTCAGGGAAG -3’<br>5’- TGCCGATATCGAATTCCTGACTCGTTTCGGTTTCCAAC -3’
Human BIM	5’-CCTCATCCCTCCAAGAAAGGAGC-3’<br>5’-CAACCACTGAGGATCCTTGCCTTATCTGTTGGGCTTTG-3’<br>5’-TGCCGATATCGAATTCCCAGTCCTTTACCACAGTTTACCAG-3’
Human NOXA	5’-GGACTGGTTCAGGAGCATCCCGG-3’<br>5’-CAACCACTGAGGATCCTGATCGCTGCCTAGCTTGTC-3’<br>5’-TGCCGATATCGAATTCCACTACAGATCCCACCAGCC-3’
Human BID	5’-GAAAGGGCCCTCACTGTGGATGG-3’<br>5’-CAACCACTGAGGATCCGCCTTTCTATTAGCTGCAACTTC-3’<br>5’-TGCCGATATCGAATTCCTGCCAAGACAAGCACTGTG-3’
Human BAX	5’-CCAGACATACTACGAGTCTTTTG-3’<br>5’-CAACCACTGAGGATCCGTCCTGGCAGTGAAACTGAAG-3’<br>5’-TGCCGATATCGAATTCCAACAAAGCCAAGACGCCTG-3’
Human BAK	5’-CCAGACATACTACGAGTCTTTTG-3’<br>5’-CAACCACTGAGGATCCGTCCTGGCAGTGAAACTGAAG-3’<br>5’-TGCCGATATCGAATTCCAACAAAGCCAAGACGCCTG-3’
Human MULE	5’-GCTAACTCGGCTACAACATTTGG-3’<br>5’-CAACCACTGAGGATCCGGTTCACACCATTCTCCTGC-3’<br>5’-TGCCGATATCGAATTCCAGGGCACCAATAGTTCTAACTC-3’
Mouse Bclx	5’-TGGCAACCCATCCTGGCACCTGG-3’<br>5’-CAACCACTGAGGATCCTCGACTTTCTCTCCTACAAGC-3’<br>5’-TGCCGATATCGAATTCCAAGGCTCTAGGTGGTCATTCAG-3’
